# Lysophosphatidylethanolamine improves diastolic dysfunction by alleviating mitochondrial injury in the aging heart

**DOI:** 10.1016/j.jlr.2024.100713

**Published:** 2024-11-22

**Authors:** Guiwen Xu, Wei Xiao, Pengqi Sun, Yuanjun Sun, Xinyu Yang, Xiaomeng Yin, Yang Liu

**Affiliations:** 1 Institute of Cardiovascular Diseases, the First Affiliated Hospital of Dalian Medical University, Dalian, China; 2 Department of Cardiology, the First Affiliated Hospital of Dalian Medical University, Dalian, China

**Keywords:** aging, mitochondria, diastolic dysfunction, lipid, lysophosphatidylethanolamine

## Abstract

Diastolic dysfunction in aging mice is linked to mitochondrial abnormalities, including mitochondrial morphology disorders and decreases in membrane potential. Studies also show that aberrant mitochondrial lipid metabolism impairs mitochondrial function in aging cardiomyocytes. Our lipidomic analysis revealed that phosphatidylethanolamine (PE) levels were significantly decreased in aging myocardial mitochondria. Here, we investigated whether a reduction in PE levels in myocardial mitochondria contributes to mitochondrial injury as well as HFpEF pathogenesis and whether modulation of PE levels could ameliorate aging-induced HFpEF. Echocardiography was used to assess cardiac diastolic function in adult and aging mice treated with lysophosphatidylethanolamine (LPE) or saline. Mitochondrial morphologies from tissue samples were evaluated by transmission electron microscopy (TEM), while mitochondrial membrane potential and reactive oxygen species (ROS) levels were assessed using JC-1, MitoSOX, and DCFH-DA detection assays. We performed GO enrichment analysis between adult and aging mice and discovered significant enrichment in transcriptional programs associated with mitochondria and lipid metabolism. Also, mitochondrial PE levels were significantly decreased in aging cardiomyocytes. Treatment with LPE (200 μg/kg) significantly enhanced PE content in aging mice and improved the structure of mitochondria in cardiac cells. Also, LPE treatment protects against aging-induced deterioration of mitochondrial injury, as evidenced by increased mitochondrial membrane potential and decreased mitochondrial ROS. Furthermore, treatment with LPE alleviated severe diastolic dysfunction in aging mice. Taken together, our results suggest that LPE treatment enhances PE levels in mitochondria and ameliorates aging-induced diastolic dysfunction in mice through a mechanism involving improved mitochondrial structure and function.

Heart failure (HF) with preserved ejection fraction (HFpEF) has become the most common form of heart failure in the elderly (1) and is associated with high morbidity and mortality. Indeed, aging is an independent risk factor of HFpEF (2, 3). Cardiac aging is accompanied by a decline in mitochondrial function, resulting in increased ROS production and dysfunctional energy metabolism (4). This, in turn, contributes to the deterioration of organs over time (5, 6). Whether there is a direct link between mitochondrial metabolism and mitochondrial dysfunction in aging mice remains poorly understood.

Mitochondria are double-membrane organelles comprising distinct lipids that include phosphatidylcholine (PC), phosphatidylethanolamine (PE), cardiolipin (CL), phosphatidylserine (PS), phosphatidic acid (PA), phosphatidylinositol (PI), and more. Membrane lipids play a key role in maintaining proper structural integrity and activity of membrane proteins, including those proteins involved in oxidative phosphorylation and electron transport chain functions (7). Additionally, mitochondria are the sites for fatty acid oxidation (8), and it has been shown that declining mitochondrial function in aging cardiomyocytes represses fatty acid oxidation (9). Lipid metabolism in aging cells has been reported in several studies (10, 11, 12); however, the significance of lipid metabolism within mitochondria and how this function influences aged cardiomyocytes remains unclear.

In the present study, we performed RNA-seq analysis of murine ventricles and found that 22-month-old mice with reduced diastolic function exhibited a gene expression profile distinct from 3-month-old mice. Moreover, when compared with adult mice, hearts from aged mice had significant alterations in the expression of genes associated with mitochondrial dysfunction and lipid metabolism. Furthermore, we found the PE levels were significantly decreased in aged mitochondria and this was associated with elevated levels of molecular markers of mitochondrial injury. On the basis of these findings, we surmise that reductions in PE levels for myocardial mitochondria can contribute to mitochondrial injury as well as HFpEF pathogenesis and that interventions that enhance PE production may ameliorate aging-induced HFpEF.

## Method

### Animal experiments

All animal experimental procedures were approved by the Animal Care and Use Committee of Dalian Medical University (AEE22063). C57BL/6J male mice were procured from Vital River Laboratory (China). All mice were housed under standard laboratory conditions, including 12 h light/dark cycles and free access to chow and water. Mice were divided into two groups: 3-month-old mice were defined as the adult group, and 22-month-old mice were classified as the aging group. For treatment groups, 3-month-old mice and 19-month-old mice were intraperitoneally injected with lysophosphatidylethanolamine (LPE, 100, 200, 400, and 800 μg/kg, 846,725, Avanti Polar Lipids) or saline once every two days for 3 months, so as to evaluate system-level metabolic effects (such as body weight, adipose weight, glucose tolerance, insulin tolerance, and liver histology), cardiac function and the ratio of heart weight to tibial length; as well as LPE supplementation of 19-month-old mice when they reached 22 months of age (13). At the end of the experimental period, serum was harvested to measure the level of alanine transaminase (ALT) and aspartate aminotransferase (AST) using automated and standardized procedures (Roche Hitachi 917/747), in order to assess liver damage.

### Echocardiography

Echocardiograms were achieved using a Vevo 1100 ultrasound machine (VisualSonics, Canada) equipped with a 30 MHz transducer. Mice were anesthetized by continuously ventilated isoflurane (0.5%–1%, 792,632, Sigma-Aldrich), then chest hair was removed with gel and the mice were placed on heating pads. Left ventricular (LV) internal diameter at end-diastole and end-systole (LVIDd, LVIDs), anterior wall (AWd/AWs), and posterior wall (PWd/PWs) thickness of end-diastole and end-systole were all measured from their parasternal short-axis views. The E/A ratio (early to late mitral inflow velocity ratio) was measured from the apical four-chamber view by pulsed Doppler echocardiography, which was used to assess diastolic function. LV ejection fraction (LVEF) was calculated by biplane Simpson’s formula to evaluate systolic function. Data were analyzed offline in a blinded fashion using VevoLab software (VisualSonics).

### Glucose tolerance test (GTT) and insulin tolerance test (ITT)

GTT was performed on mice fasted for 16 h, following which an intraperitoneal injection of glucose (2 g/kg body weight) was delivered before blood glucose measured at 0, 30, 60, 90 and 120 min after injection. ITT was performed on mice fasted for 6 h by giving an intraperitoneal injection of insulin (0.75 units/kg body weight, Humulin R). Blood glucose was measured at 0, 15, 30, and 60 min after injection.

### Histological analysis

Dissected heart and liver tissues were fixed in 4% paraformaldehyde, embedded in paraffin before 5 μm sections were prepared from these blocks of tissue using a microtome (RM2125RTS, Leica). Masson staining was used to evaluate the extent of fibrosis in the cardiac and hepatic tissues according to the manufacturer’s instructions (Masson Kit, G1340). The percentage of fibrosis was quantified using ImageJ software. To evaluate cardiac hypertrophy, cardiac sections were incubated with Alexa Fluor™ 555 conjugated with wheat germ agglutinin (WGA, 1:1000, W32464, Invitrogen). Images were acquired using a fluorescence microscope (NI-U, Nikon) and 50 randomly selected cardiomyocytes were analyzed to measure their cross-sectional area (CSA) using ImageJ software.

### Oil red O staining

Oil Red O staining was performed to evaluate lipid accumulation using 5 μm-thick frozen sections prepared from liver tissue blocks embedded in optimal cutting temperature (OCT, 4583, Sakura) medium. The sections were washed in 60% isopropanol for 5 min and then incubated with the Oil Red O working solution (saturated oil red O solution mixed at a 3:2 ratio with distilled water, O9755, sigma) for 20 min. This procedure was followed by sequentially washing with 60% isopropanol and water. Then, the sections were mounted with glycerol gelatine (G1402, Servicebio) for imaging.

### Transmission electron microscopy analysis

Cardiac (left ventricle) tissue samples from six individuals within each treatment group were quickly isolated and cut into 1–2 mm^3^ cubes, washed with saline buffer, and fixed in 2.5% glutaraldehyde (G6257, Sigma) diluted in 0.1 M phosphate buffer (pH 7.4, P4474, Sigma) overnight at 4°C, then post-fixed with 4% osmium tetroxide (20,816-12-0, Sigma) in phosphate buffer for 2 h at room temperature. Next, the samples were dehydrated with gradient ethanol, and embedded in agar. Heart tissue was cut into sections at thicknesses of between 50 nm and 70 nm. After staining with 2% uranium acetate saturated alcohol solution (541-09-3, Syntechem) and 2% lead citrate solution (512-26-5, Aladdin), the structures of mitochondria were observed under a transmission electron microscope (TEM, JEOL-1200EX). Ultrastructural damage was quantified based on the scoring method published by Flameng and colleagues (14). Mitochondria were delineated using Image J software for calculation of mitochondria area, perimeter, Feret's diameter (defined as the longest distance between any two points in the mitochondria), and the ratio of cristae-covered areas relative to the whole mitochondrial area. For each sample, 5 field views were randomly selected and approximately 20 individual mitochondria were selected per magnification field view to calculate each parameter.

### Isolation and culture of cardiomyocytes

A simplified, Langendorff-free method was used for the isolation of viable cardiomyocytes from the hearts of adult and aging mice (15). Fresh buffer and media for experiments with each mouse were prepared as follows: 30 ml EDTA Buffer (NaCl 130 mM, KCl 5 mM, NaH_2_PO_4_ 0.5 mM, HEPES 10 mM, Glucose 10 mM, 2,3-butanedione monoxime (BDM) 10 mM, Taurine 10 mM and EDTA 5 mM, pH 7.8), 20 ml Perfusion Buffer (NaCl 130 mM, KCl 5 mM, NaH_2_PO_4_ 0.5 mM, HEPES 10 mM, Glucose 10 mM, BDM 10 mM, Taurine 10 mM and MgCl_2_ mM, pH 7.8), 60 ml Collagenase Buffer (Collagenase II 0.5 mM, Collagenase IV 0.5 mM and Protease Type XIV 0.05 mM) and 10 ml Stop Buffer (containing 95% Perfusion buffer and 5% sterile FBS), Culture Media (M999, 0.1% Bovine serum albumin, 1× ITS (insulin, transferrin, selenium), 1× CD lipid (chemically defined lipid concentrate) and 10 mM BDM), and Calcium Reintroduction Buffer with three different Ca^2+^ concentrations (0.34 mM, 0.68 mM and 1.02 mM), which were prepared by mixing different proportions of Perfusion Buffer and Culture Media. All reagents were obtained from Millipore Sigma unless otherwise noted.

Mice were anesthetized, following which the chest cavity was opened and the heart exposed. The descending aorta was then severed, and the right ventricle was flushed immediately with 7 ml EDTA Buffer. The ascending aorta was clamped with Reynolds forceps, followed which sequential injections of 10 ml EDTA Buffer, 3 ml Perfusion Buffer, and 30 ml preheated Collagenase Buffer were introduced into the left ventricle. Then, separated heart chambers were gently sliced into 1 mm pieces. After sufficient enzymatic digestion, 5 ml of Stop Buffer was added. The suspension was then passed through a 100 μm mesh filter (CLS431752, Sigma) to separate and remove undigested tissue debris. Cells then underwent 4 consecutive rounds of gravity settling in 20 min, using 3 intermediate Calcium Reintroduction Buffers to gradually restore calcium concentration to physiological levels. The resulting cardiomyocytes were seeded on plates coated with laminin (5 μg/ml, A29248, Gibco) and cultured in prewarmed culture medium at 37°C in a 5% CO_2_ incubator.

### Mitochondrial stress test

Mitochondrial stress tests were performed in an XFe96 extracellular flux analyzer (Agilent). AC16 cells or isolated cardiomyocytes from adult and aging mice with or without LPE supplementation were cultured at a density of 1 × 10^4^ and 2 × 10^3^, respectively, for 24 h in Seahorse 96-well XF cell culture microplates coated with laminin (10 μg/ml, 23017035, Gibco). In parallel, XFe96 sensor cartridges were hydrated overnight with sterile water in an incubator at 37°C. Before measurement, the cell culture medium for each well of cells was replaced with XF assay medium (XF Base Medium 97 ml, 1.0 M Glucose Solution 1 ml, 100 mM Pyruvate Solution 1 ml, and 200 mM Glutamine Solution 1 ml, pH = 7.4). Both sensor and cell plates were incubated in a CO_2_-free incubator at 37°C for 1 h before the assay. Seahorse injection ports were loaded with a final concentration of 1 μM oligomycin (port A), 1 μM carbonyl cyanide trifluoromethoxy phenylhydrazone (FCCP, port B), and 1 μM rotenone/antimycin A (port C), and the basal respiration (initial oxygen consumption rate) and maximal respiration (oxygen consumption following FCCP addition) measurements were recorded. All the reagents were provided in the Seahorse XF Cell Mito Stress Test Kit (103,015-100, Agilent). Data were analyzed using Agilent Seahorse Wave software.

### Mitochondria isolation

Mitochondria were separated from the heart using a commercially available kit (KTP4004, Abbkine). Fresh hearts were excised and washed thoroughly with ice-cold 0.9% NaCl solution. Then, 100 mg of cardiac tissue was cut into small pieces, treated with 1 ml trypsin (0.25 mg/ml), and incubated on ice for 8 min, and then the supernatant was discarded. Next, the samples were homogenized with ice-cold lysis buffer. Intact mitochondria were isolated through the process of differential centrifugation. Homogenized samples were centrifuged at 600 *g* for 10 min twice, and then the supernatant was collected and centrifuged at 11,000 *g* at 4°C for 10 min. The protein concentration of mitochondria for each sample was determined spectrophotometrically. Purified mitochondria were resuspended in Storage Buffer and stored at −80°C until use.

### Transcriptomics analysis

Heart tissues were obtained from 3-month-old mice and 22-month-old mice. Heart tissues were collected and analyzed from mice using a sample preparation, nucleic acid extraction, sequencing, and detection protocol for transcriptomic analysis that was recommended by the commercial service provider, MetWare. Through an Illumina Hiseq 4000 sequencing platform, the cDNA libraries were sequenced for transcriptomics. Between two groups (adult vs. aging mice), differentially expressed genes were determined by absolute Log2FC ≥ 1, adjust *P* value < 0.05. GeneOntology (GO) enrichment analysis was utilized to reveal significant enrichment for transcriptional programs between adult and aging mice.

### Lipid sample preparation and lipidomic assays

Lipids from the cardiac mitochondria of such samples from adult and aging mice were extracted using a modified methyl tert-butyl ether (MTBE) lipid extraction method (16). Internal standards including SPLASH Mix (330,707, Avanti Polar Lipids) and Cardiolipin Mix I (LM6003, Avanti Polar Lipids) were added to the samples before extraction. Lipids were extracted by mixing MTBE/methanol/water (10:3:2.5, v/v/v), and the upper phase was collected. The organic phases were dried in a vacuum centrifuge, dissolved in 200 μl of CHCl3/methanol/water (60:30:4.5, v/v/v), and stored at −80°C. For lipid analysis, liquid chromatography-mass spectrometry (LC-MS) was performed on an Agilent 6530 high-performance liquid chromatography-quadrupole time-of-flight mass spectrometer. Lipid molecules with VIP > 1 and *P*-value < 0.05 were considered as differential lipids.

### PE content determination

Mitochondria of four groups (adult and aging mice supplemented with LPE or saline) were extracted according to the method described above. In order to investigate the effect of exogenous LPE supplementation, PE assay kit (MAK361, Merck) was used to determine the PE content of cardiac mitochondria. Briefly, mitochondria were homogenized in 5% Triton X-100, and then heated to 80°C to solubilize all lipids. Next, 10 μl of each sample (or standard) was added to wells of a 96-well plate together with a Working Reagent Mix. The fluorescence intensity from each well was measured with a microplate reader (M1000PRO, TECAN) at excitation/emission wavelengths of 535/587 nm, respectively.

### Mitochondrial membrane potential

Mitochondrial membrane potential was measured using a JC-1 MitoMP Detection Kit (Dojindo Laboratories, MT09) according to the manufacturer's instructions. Isolated cardiomyocytes were seeded onto the bottom of a glass cell culture dish and cultured in a 5% CO_2_ incubator at 37°C. To measure mitochondrial membrane potential, JC-1 working liquid prepared and then incubated with the cells for 30 min. Next, the supernatant was removed, and cells were then washed twice with Hank’s balanced salt solution, and the Imaging Buffer solution was added. JC-1 monomers and polymers were then examined under a confocal microscope (DMi8, Leica) at excitation wavelengths of 488 nm and 561 nm, respectively. Loss of mitochondrial membrane potential was represented by changes in JC-1 fluorescence from red (JC-1 polymer, energized mitochondrial membrane potential) to green (JC-1 monomers, lower mitochondrial membrane potential). Image J was used to analyze the ratio of red to green fluorescence.

### Mitochondrial ROS detection with MitoSOX

Mitochondrial ROS/superoxide within cells were stained with MitoSOX Deep Red (MT14, Dojindo Laboratories) and analyzed. To do this, a freshly prepared working solution containing 10 μmol/L MitoSOX Red was incubated with cardiomyocytes for 30 min at 37°C. Cells were then washed twice with phosphate-buffered saline (PBS) before image acquisition, using under a confocal microscope at the excitation wavelength of 540 nm. Image J software was used to evaluate the average fluorescent intensity. Superoxide selectively oxidizes MitoSOX Deep Red in the mitochondria, resulting in the production of a purple fluorescence signal.

### Intracellular ROS detection by DCFH-DA

The intracellular ROS levels were detected through an assay employing the highly sensitive dye known as 2,7-dichlorofluorescein diacetate (DCFH-DA, R252, Dojindo Laboratories). This DCFH-DA dye was diluted to a 1:1000 working solution with loading buffer and incubated with the cardiomyocytes for 30 min at 37°C in a 5% CO2 incubator. Next, the cardiomyocytes were washed with PBS, and confocal microscopy analysis was performed to detect signals emanating from an excitation wavelength of 488 nm. The average fluorescence intensity from captured images was analyzed by Image J.

### Cell culture and transfection

Human cardiomyocyte AC16 cells were cultured in DMEM cell culture medium (11965092, Gibco) containing 10% FBS (10100147C, Gibco) and 1% penicillin/streptomycin (TMS-AB2-C, Sigma-Aldrich) at 37°C with 5% CO_2_. To induce cellular senescence, AC16 cells were treated with 20 g/L D-galactose (D-gal, V900922, Sigma) for 48 h. The siRNA oligonucleotides for knockdown of phosphatidylserine decarboxylation (*PISD*) to reduce mitochondrial PE synthesis in cultured cells have been described (17). The siRNA sequences for *PISD* (5′-AAGUAGAUGCGAAUGGAGCCC-3′) and control siRNA (5′-ACGUGACACGUUCGGAGAATT-3') were designed and synthesized by Hanbio Biotechnology (Shanghai, China). Transfections with 100 nmol/L siRNA molecules were performed with GP-transfect-mate (G04009, GenePharma) according to the manufacturer's instructions. To assess the effects of LPE treatment, AC16 cells were transfected with control siRNA or *PISD* siRNA and immediately supplemented with and without LPE 18:0 (100 μM). Cells were processed for subsequent analyses 2 days after siRNA transfection.

### Senescence-associated β-galactosidase (SA-β-gal) staining

SA-β-gal staining was performed according to the manufacturer’s protocol using SA-β-gal staining kit (G1580, Solarbio). Briefly, after treatment with D-gal to induce senescence, AC16 cells were washed with PBS and fixed with 4% paraformaldehyde for 15 min at room temperature. Following three washes with PBS, cells were incubated in SA-β-gal staining solution overnight in a 37°C dry incubator (no CO2 condition). Senescent cells were identifiable by light microscopy and were stained green, and the percentages of SA-β-Gal positive cells were then calculated for each well of cells across all treatment conditions.

### Real-time PCR analysis

Cardiac tissues were collected to evaluate the expression of genes related to mitochondrial dynamics and metabolism by real-time PCR. The mitochondrial dynamics-related genes include optic atrophy 1 (*Opa1*), mitofusin 1 (*Mfn1)*, mitofusin 2 (*Mfn2)*, fission 1 (*Fis1*) and dynamin-related protein 1 (*Drp1*). Mitochondrial tricarboxylic acid (TCA) cycle isocitrate genes involve isocitrate dehydrogenase 3 (NAD+) gamma (*Idh3g*), succinyl-CoA ligase (*Suclg1*) and malate dehydrogenase 2 (*Mdh2*). Mitochondrial oxidative phosphorylation (OXPHOS) related genes include NADH-ubiquinone oxidoreductase core subunit V1, V2 (*Ndufv1*, *Ndufv2*), NADH-ubiquinone oxidoreductase Fe-S protein 1, 2, 3, 7, 8 (*Ndufs1*, *Ndufs2*, *Ndufs3*, *Ndufs7 and Ndufs8*), ubiquinol-cytochrome c reductase, Rieske iron-sulfur polypeptide 1 (*Uqcrfs1*), ATP synthase peripheral stalk-membrane subunit b (*Atp5pb*) and ATP synthase, H^+^ transporting, mitochondrial F0 complex subunit C2 (subunit 9) (*Atp5g2*). PE is primarily synthesized through the cytidine diphosphate (CDP)-ethanolamine pathway (Kennedy pathway) and mitochondrial PS decarboxylase (PSD) pathway, however it can also be produced via the acylation of LPE. Genes involved in the Kennedy pathway of PE biosynthesis include ethanolamine Kinase 1 (*Etnk1*), phosphoethanolamine cytidylyltransferase (*Pcyt2*), and Selenoprotein 1 (*Selenoi*). PSD pathway-related genes include *Pisd*. Genes related to the acylation of LPE include acyl-CoA transferase enzymes membrane bound O-acyltransferase domain containing 1 (*Mboat1*), lysophosphatidylcholines acyltransferase 3 (*Lpcat3*), and lysophosphatidylcholines acyltransferase 4 (*Lpcat4*). Additionally, PE is converted to PS via PS-synthase-2 (*Ptdss2*) catalyzed base-exchange reactions. While the level of mitochondrial PS, which is the precursor of PE, is determined by PS-synthase-1 (*Ptdss1*), Ptdss2, and PRELI Domain Containing 3B (*Prelid3b*). AC16 cells were harvested and processed for the analysis of gene expression levels for *PISD*, *DRP1*, *FIS1*, *OPA1*, *MFN1* and *MFN2*, using real-time PCR. Total RNA was extracted from hearts using the TRIzol reagent (15596018, Thermo Fisher Scientific). cDNA was synthesized using a cDNA reverse transcription kit (RR047 A, Takara) according to the manufacturer’s protocol. Real-time quantification experiments were performed using SYBR-green PCR assays and Applied Biosystems 7500 system detection device (Thermos fisher Scientific, USA). Relative gene expression levels were normalized to beta-actin (*Actb*) and calculated using the comparative threshold cycle method. The sequences of gene specific primers are listed in Supplemental Tables S1 and S2.

### Quantification and statistical analysis

Variables were presented as mean ± standard error of the mean (SEM). Normal distribution and homogeneity of variance were both tested for all datasets. Data comparisons between two groups were performed using unpaired Student's t-tests (for the normal distribution and equal variances), Welch's *t* test (for unequal variances), and Mann–Whitney's test (for non-compliance with a normal distribution). For multiple groups, univariate comparisons were performed by one-way analysis of variance (ANOVA) followed by Tukey’s post-hoc tests (for normal distribution and equal variances), Brown-Forsythe and Welch ANOVA tests followed by Dunnett’s T3 post-hoc test (for unequal variances) and Kruskal-Wallis tests followed by Dunn’s post-hoc test (for non-compliance with the normal distribution), while bivariates were analyzed by two-way ANOVA test using Geisser-Greenhouse correction, followed by Bonferroni post-hoc test. *P* values below 0.033 were considered significant as follows: *P* < 0.033 (∗), *P* < 0.002 (∗∗), *P* < 0.001 (∗∗∗). Statistical analyses were performed using GraphPad Prism 9 (GraphPad Software).

## Results

### Cardiac diastolic dysfunction and structural remodeling in aging (22-month-old) mice

**Fig. 1:**
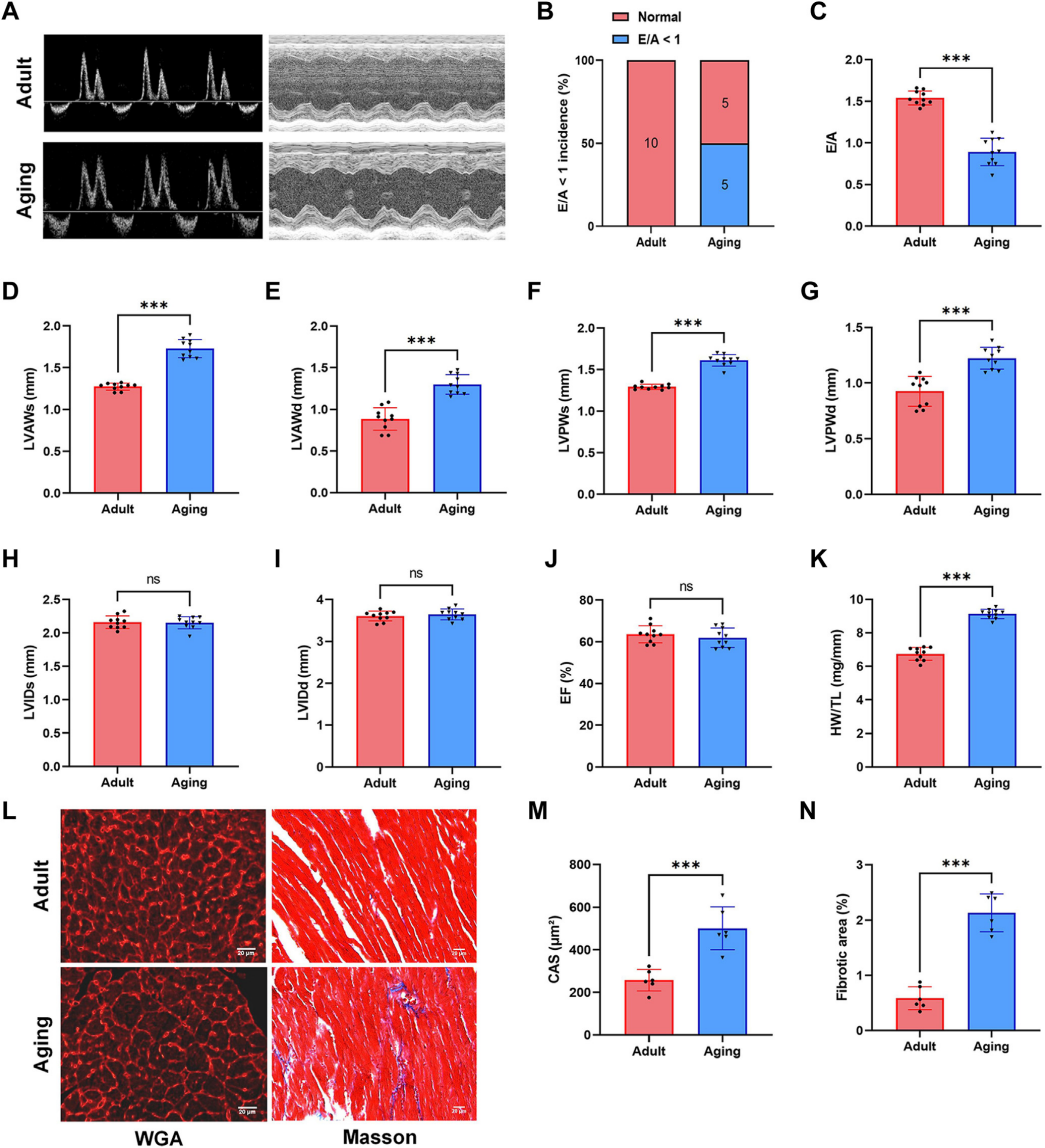
Cardiac diastolic dysfunction and structural remodeling in aging (22-month-old) mice. Representative left ventricular M-mode images and pulse-wave Doppler images of adult and aging mice (A). The proportion of mice with E/A < 1 (B). Assessment of cardiac function by echocardiography: E/A (C), LVAWs (D), LVAWd (E), LVPWs (F), LVPWd (G), LVIDs (H), LVIDd (I) and EF% (J). Quantitation of HW to TL in adult and aging mice (K). Representative images of wheat germ agglutinin (WGA) staining for cardiomyocyte cross-sectional area measurements (CSA), and Masson staining for ventricular fibrosis (L). Quantification of cardiomyocytes CSA and fibrotic area (M and N). E/A: the ratio of early diastolic transmitral flow velocity to late diastolic transmitral flow velocity, LVAWs/d: left ventricular anterior wall at end-systole/end-diastole, LVPWs/d: left ventricular posterior wall at end-systole/end-diastole, LVIDs/d: left ventricular internal dimension at end-systole, EF: ejection fraction, HW: heart weight, TL: tibia length. All data were analyzed using a Student’s *t* test. ∗*P* < 0.033, ∗∗*P* < 0.002, ∗∗∗*P* < 0.001. Statistical analysis was conducted using a Student's *t* test. Samples sizes were n = 10 per group except for the WGA staining and Masson staining analysis (n = 6 per group).

Echocardiography was performed to investigate the cardiac systolic and diastolic functions in adult and aging mice (Fig. 1A). The incidence of diastolic dysfunction in 22-month-old mice was 50%, as measured by decreased E/A (E/A < 1) values (Fig. 1B), and the E/A ratio was significantly reduced by 42.2% in aging mice compared to adult mice (Fig. 1C). Also, LV wall thickness was increased in the aging hearts compared to adult hearts, measured as LVAWs/d and LVPWs/d (Fig. 1D–G). There were no significant differences in LVIDs/d and EF% features between adult and aging mice (Fig. 1H–J). Furthermore, the ratio of heart weight (HW) to tibia length (TL) was increased in the aging group (Fig. 1K). Measurements of cardiomyocyte CSA were significantly increased in aging mice relative to the adult mice as indicated by WGA staining (Fig. 1L, M). Cardiac fibrosis was also notably increased in aging mice (Fig. 1L, N).

### Abnormal mitochondrial respiration and morphology, and reduced mitochondrial membrane potential in aging hearts

**Fig. 2:**
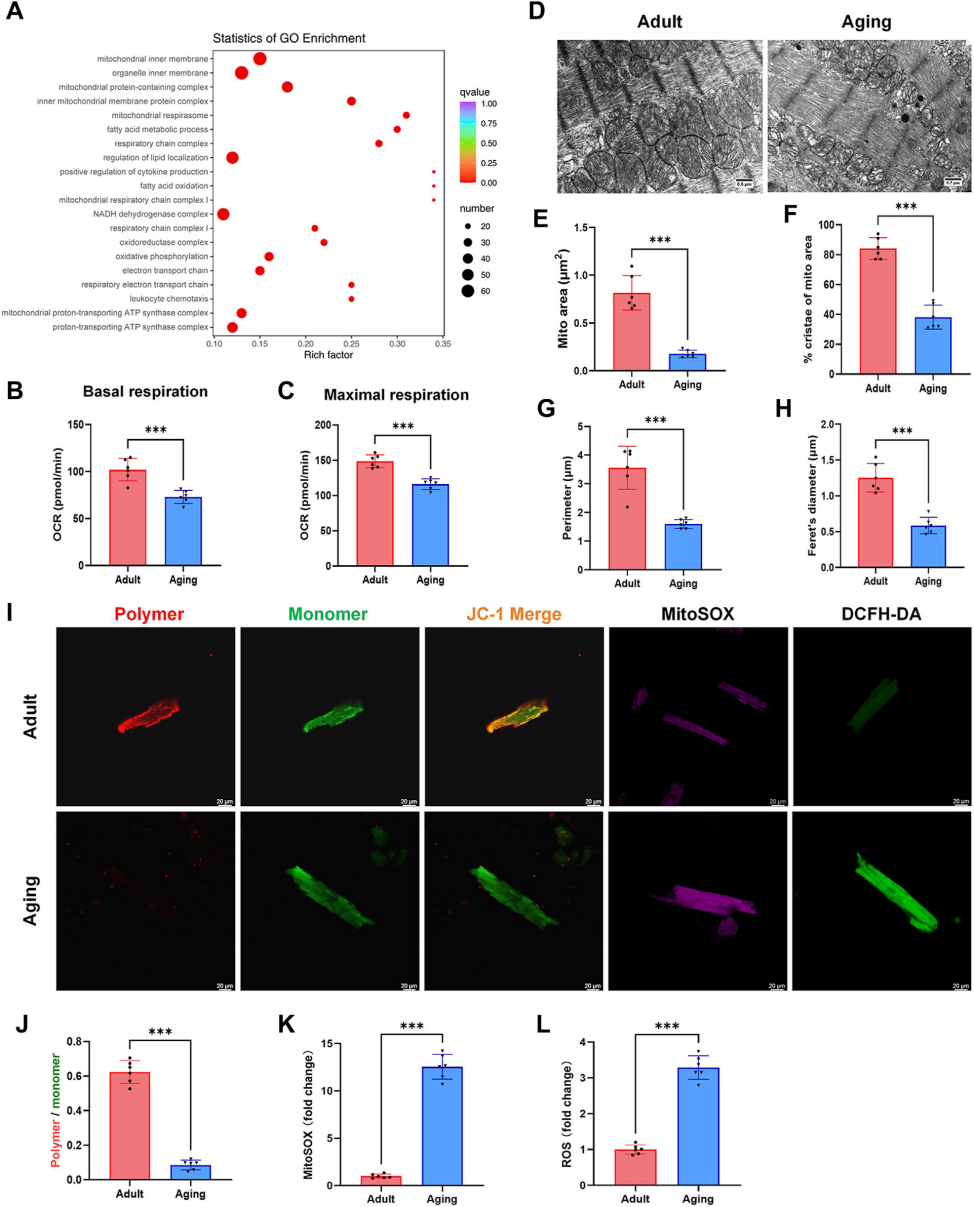
Abnormal mitochondrial respiration and morphology, and reduced mitochondrial membrane potential in aging hearts. GeneOntology (GO) enrichment analysis of differential genes identified between adult and aging mice heart tissue samples (A). Basal respiration (B) and maximal respiration (C) for cardiomyocytes were quantified from the Mito Stress Test. Representative transmission electron microscope (TEM) images of the mitochondria in adult and aging groups (D). Mitochondrial parameters were analyzed to quantify their morphology: mitochondria area (μm^2^) (E), the ratio of cristae-covered area to the whole mitochondria area (F), perimeter (μm) (G), and Feret's diameter (longest axis of mitochondria) (μm) (H). Representative images of cardiomyocytes stained with JC-1 (*red* fluorescence for JC-1 polymer, *green* fluorescence for JC-1 monomers), MitoSox (*purple* fluorescence), and DCFH-DA (*green* fluorescence) for mitochondrial membrane potential, mitochondrial reactive oxidative species (ROS) and intracellular ROS, respectively (I). Quantitative analysis of mitochondrial membrane potential, expressed as a ratio of JC-1 polymer to JC-1 monomer fluorescence intensity (J). Quantitative analysis of fluorescence intensity for MitoSox (K) and DCFH-DA (L). All data were analyzed using a Student’s *t* test. ∗*P* < 0.033, ∗∗*P* < 0.002, ∗∗∗*P* < 0.001. Samples sizes were n = 6 per group except for the transcriptomics-related analysis (n = 4 per group).

We carried out RNA-seq of adult and aging heart tissue samples. The gene expression landscapes for the two groups are provided in Supplemental Table S3. From this, we performed GO enrichment analysis and discovered that the top 20 enriched pathways were related to mitochondrial structure, mitochondrial functions, and lipid metabolism, such as mitochondrial inner membrane, mitochondrial protein-containing complex, inner mitochondrial membrane protein complex, mitochondrial respirasome, fatty acid metabolic process, regulation of lipid localization, fatty acid oxidation, and mitochondrial respiratory chain complex I (Fig. 2A).

Seahorse XFe96 Cell Mito Stress Test analyses were performed to assess the oxygen consumption rate (OCR) in isolated adults and aging cardiomyocytes. As shown, mitochondrial basal respiration and maximal respiration were both significantly decreased in aging mice, compared to adult mice (Fig. 2B, C). In parallel, the ultrastructure of mitochondria within cells of the adult and aging male mice hearts were investigated by TEM (Fig. 2D). As shown, while mitochondria in the cardiomyocytes of adult mice appeared healthy and normal, exhibiting well-arranged cristae, mitochondria from samples of the aging mice group showed reduced mitochondrial surface area, as well as reduced cristae densities, perimeters and Feret's diameter (Fig. 2E–H). Next, the functional integrity of mitochondria from isolated viable cardiac myocytes from the adult and aging mouse heart was assessed by JC-1 staining (Fig. 2I). Compared with the adult group, the JC-1 polymers to monomers fluorescence ratio was decreased in readings from cardiomyocytes of aging mice (Fig. 2J). Moreover, MitoSOX and DCFH-DA staining were used to measure mitochondrial and cytoplasmic ROS in the adult and aging cardiomyocytes (Fig. 1I), showing that both mitochondrial and cytoplasmic ROS levels were significantly increased in aging cardiomyocytes (Fig. 2K, L).

### Identification and enrichment analysis of differential lipid metabolites

**Fig. 3:**
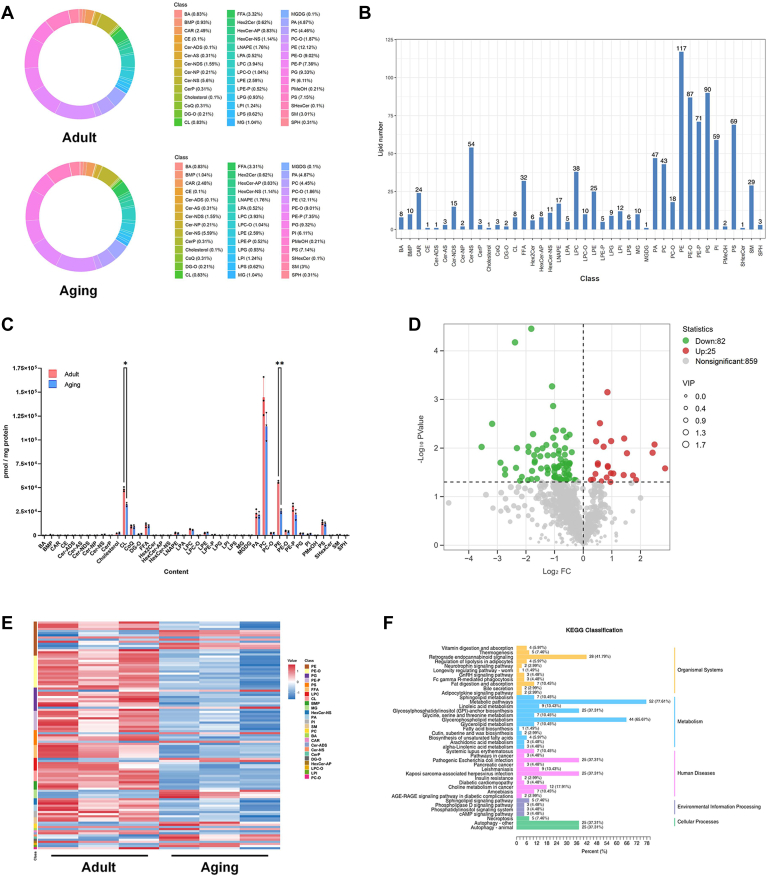
Identification and enrichment analysis of differential lipid metabolites. Circular diagram of lipid subclass composition for lipids in cardiac mitochondria between adult and aging groups (A). Bar chart showing the number of lipids contained in each of the detected lipid subclasses (B). Bar chart for content of lipid subclasses (C). Volcano plot showing differential lipid metabolites in cardiac mitochondria (D). Heat map of differential lipid metabolites by classification, red indicates up-regulated lipid molecules and blue indicates down-regulated ones (E). KEGG pathway enrichment analysis for differential lipid molecules (F). All data were analyzed using Student’s t-tests. ∗*P* < 0.033, ∗∗*P* < 0.002. Sample sizes were n = 3 per group.

We carried out an LC-MS lipidomic analysis and detected 966 lipid species in mitochondria. In total, 42 lipid subclasses were detected in the adult and aging group (Fig. 3A). Of these 42 lipid subclasses, the most abundant lipid species was PE (117 lipid molecules), followed by phosphatidylglycerols (PG), ether-linked PE (PE-O) and PE plasmalogens (PE-P) with 90, 87 and 71 lipid molecules, respectively (Fig. 3B). The content of 42 lipid subclasses was analyzed in the cardiac mitochondria of adult and aging mice. The results showed that PC, PE, and CL accounted for the majority of the total lipid content in the mitochondria of heart tissue, while PE content was significantly reduced in aging mice (Fig. 3C).

Compared with the mitochondria of adult mice, the relative abundance of 107 lipid molecules was significantly different in samples analyzed from the aging mice group, including 25 up-regulated and 82 down-regulated molecules (Fig. 3D). All the significantly differential lipid metabolites could be categorized into 25 chemical classes, with the vast majority belonging to the PE, PE-O and PG subclasses (Fig. 3E). We further explored the potential function of the differential lipid metabolites by employing KEGG enrichment analysis and found that differential lipid molecules were mainly enriched in the metabolic pathways, glycosylphosphatidylinositol (GPI)-anchor biosynthesis, glycerophospholipid metabolism and autophagy (Fig. 3F).

### Altered lipid metabolism in aging-induced mitochondria injury

**Fig. 4:**
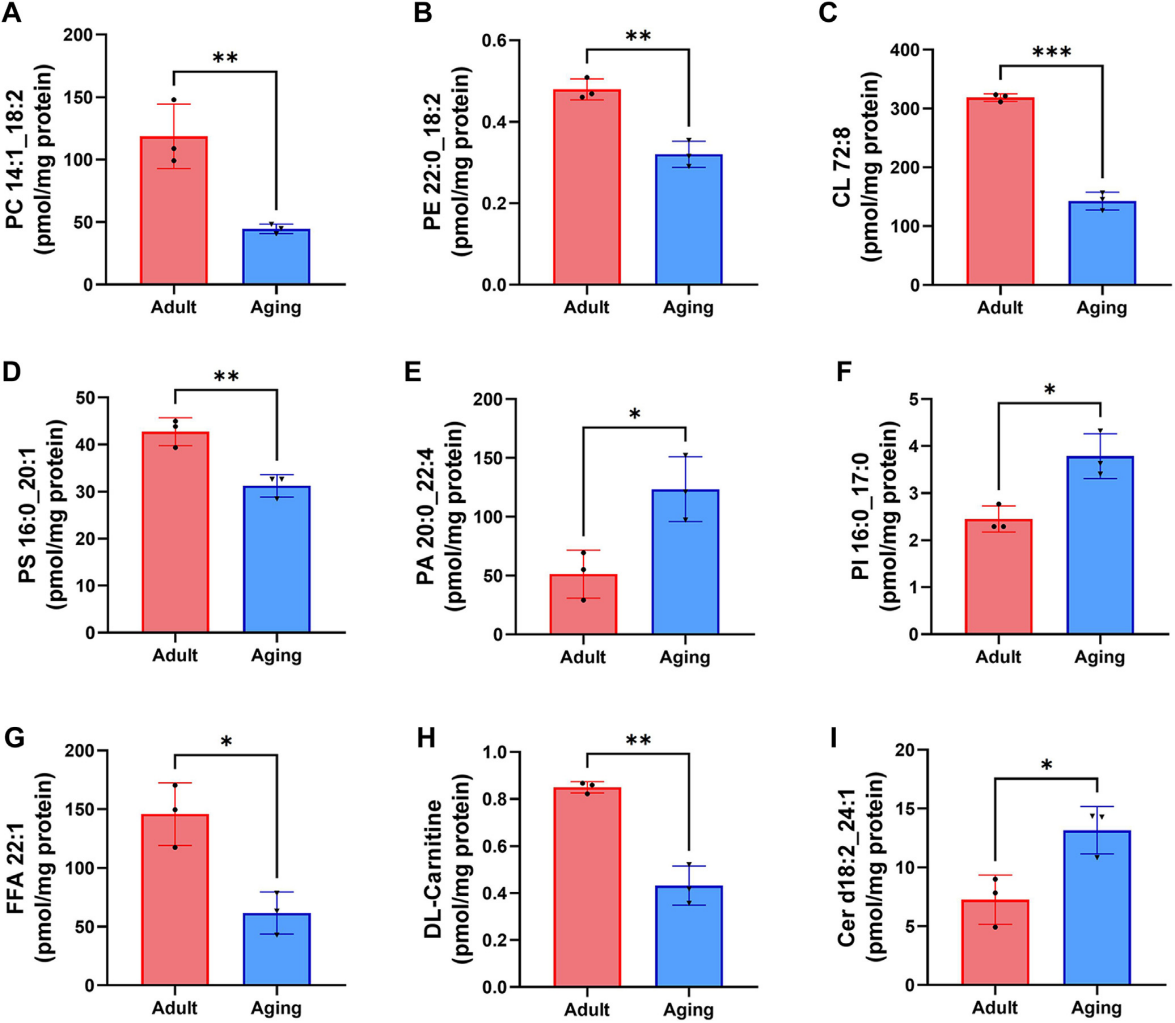
Altered lipid metabolism in aging-induced mitochondria injury. Quantification of lipid molecule content in cardiac mitochondria between adult and aging groups: phosphatidylcholine (PC 14:1_18:2) (A), phosphatidylethanolamine (PE 22:0_18:2) (B), cardiolipin (CL 72:8) (C), phosphatidylserine (PS 16:0_20:1) (D), phosphatidic acid (PA 20:0_22:4) (E), phosphatidylinositol (PI 16:0_17:0) (F), free fatty acid (FFA 22:1) (G), DL-carnitine (H) and ceramide (Cer d18:2_24:1) (I). All data were analyzed using a Student’s *t* test. ∗*P* < 0.033, ∗∗*P* < 0.002, ∗∗∗*P* < 0.001. Sample sizes were n = 3 per group.

Phospholipids are the essential component of the mitochondrial membranes, and PC, PE, and CL are collectively recognized as the most abundant phospholipids, accounting for 40%–45%, 25%–30%, and 15%–20% of the total mitochondrial phospholipids, respectively (18, 19, 20). In the aging heart, PC (14:1_18:2), PE (22:0_18:2), CL (72:8), and PS (16:0_20:1) were conspicuously down-regulated (Fig. 4A–D), while PA (20:0_22:4) and PI (16:0_17:0) were significantly up-regulated (Fig. 4E, F). In addition to the relative abundance of lipids that constitute the mitochondrial membrane, free fatty acids (FFA), carnitine, and ceramide (Cer) were also significantly changed in aging mice hearts (Fig. 4G–I). Notably, FFA (22:1) and DL-Carnitine were decreased in the aging mice, while Cer (d18:2_24:1) was markedly increased in the aging group, as compared with the adult mice (Fig. 4G–I).

### Aging-induced Pisd reduction led to mitochondrial respiration damage and mitochondrial dysfunction in cardiomyocytes ameliorated by LPE supplementation

**Fig. 5:**
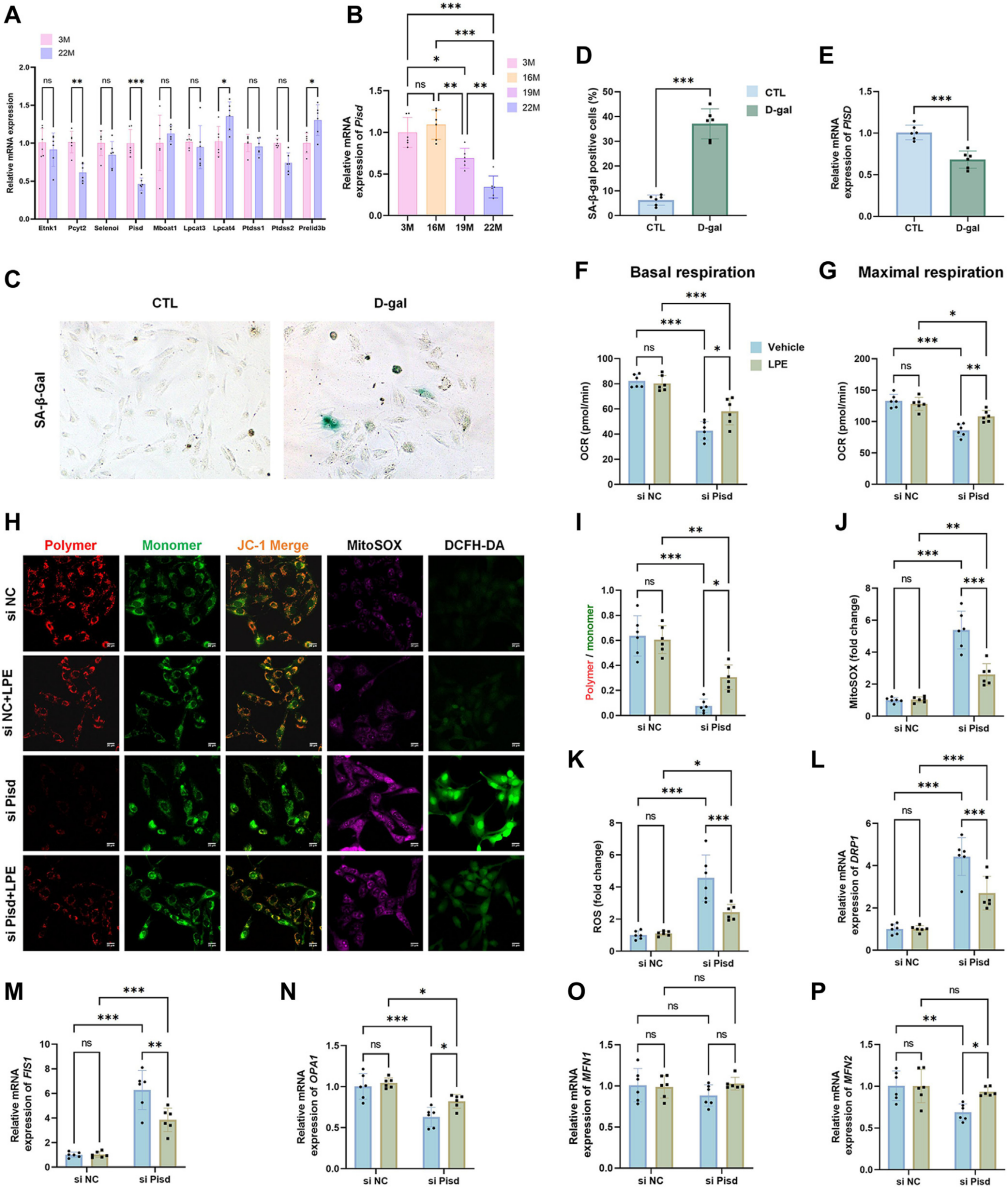
Aging-induced Pisd reduction led to mitochondrial respiration damage and mitochondrial dysfunction in cardiomyocytes were ameliorated by LPE supplementation. Quantitative analysis of mRNA levels for genes associated with the Kennedy pathway (*Etnk1*, *Pcyt2* and *Selenoi*), PSD pathway (*Pisd*), LPE acylation (*Mboat1*, *Lpcat3* and *Lpcat4*), synthesis of PS, the precursor of PE (*Ptdss1* and *Ptdss2*) and transport of PS (*Prelid3b*) (A). Quantitative analysis of *Pisd* mRNA levels in heart tissue of mice at 3M, 16M, 19M and 22M (B). Representative staining images of SA-β-Gal in control and D-gal-treated AC16 cells (C). Percentage of SA-β-Gal positive cells (D). Quantification of mRNA levels for *PISD* (E). Basal respiration (F) and maximal respiration (G) for siNC- and si*PISD*-transfected AC16 cells with or without LPE supplementation, as measured using a Mito Stress Test kit. Representative images of AC16 cells stained with JC-1 (red fluorescence for JC-1 polymer, green fluorescence for JC-1 monomers), MitoSox (purple fluorescence), and DCFH-DA (green fluorescence) (H). Quantification of mitochondrial membrane potential, evaluated as a ratio of JC-1 polymer to JC-1 monomer fluorescence intensity (I). Quantification of fluorescence intensity for MitoSox (J) and DCFH-DA (K). mRNA levels of mitochondrial fission-related genes *DRP1* (L) and *FIS1* (M), as well as fusion-related genes, *OPA1* (N), *MFN1* (O) *and MFN2* (P). Statistical analyses were performed by Student's *t* test (A, D and E), one-way ANOVA (B) and two-way ANOVA (F, G and I–P). ∗*P* < 0.033, ∗∗*P* < 0.002, ∗∗∗*P* < 0.001. Sample sizes were n = 6 per group.

Mitochondrial PE content is influenced by the main pathways for PE synthesis, including the Kennedy pathway, the PSD pathway, and the LPE acylation pathway. Moreover, mitochondrial PE content is closely related to the synthesis and transport of PS, the precursor of PE. With respect to mRNA expression levels for the key enzymes crucial to the reaction steps for the pathways mentioned herein, we found that levels of *Pcyt2*, *Pisd*, *Lpcat4*, and *Prelid3b* but not *Etnk1*, *Selenoi*, *Mboat1*, *Lpcat3*, *Ptdss1*, and *Ptdss2* were significantly altered in aging hearts compared to adult mice (Fig. 5A). Notably, mRNA level for *Pisd* (the enzyme primarily synthesized mitochondrial PE) was significantly reduced in 19-month-old and 22-month-old aging mice (Fig. 5B). To explore whether changes to *Pisd* levels were associated with the viability of aging cardiomyocytes, we subjected AC16 cells to D-gal to induce their senescence, as evaluated by SA-β-Gal staining (Fig. 5C, D). As shown, *PISD* mRNA levels were notably reduced in D-gal-treated AC16 cells compared to control (Fig. 5E). Next, we performed *PISD* knockdown in AC16 cardiomyocytes through treatment with siRNAs (denoted as si*PISD*) and this resulted in decreased mitochondrial basal respiration and maximum respiration (Fig. 5F, G). However, the reduced basal respiration and maximum respiration induced by si*PISD* treatment were alleviated by LPE supplementation (Fig. 5F, G). Moreover, the si*PISD*-transfected cardiomyocytes showed evidence of mitochondrial impairment, including reduced mitochondrial membrane potential, and increased mitochondrial and cytoplasmic ROS production (Fig. 5H–K), and these abnormalities were alleviated by LPE supplementation (Fig. 5H–K). Compared to the control, si*PISD* treatment led to increased mRNA levels for mitochondrial fission genes *DRP1* and *FIS1*, and decreased levels for mitochondrial fusion gene *OPA1* and *MFN2* (Fig. 5L–N, and P), while *MFN1* mRNA expression was comparable between two groups (Fig. 5O). Furthermore, LPE supplementation could ameliorate the abnormal mRNA levels for *DRP1*, *FIS1*, *OPA1*, and *MFN2* in si*PISD*-transfected cardiomyocytes; however *MFN1* mRNA level was not affected in this context (Fig. 5L–P).

### Metabolic phenotypes and safety characteristics of LPE exposure in treated mice

**Supplemental Figure S1:**
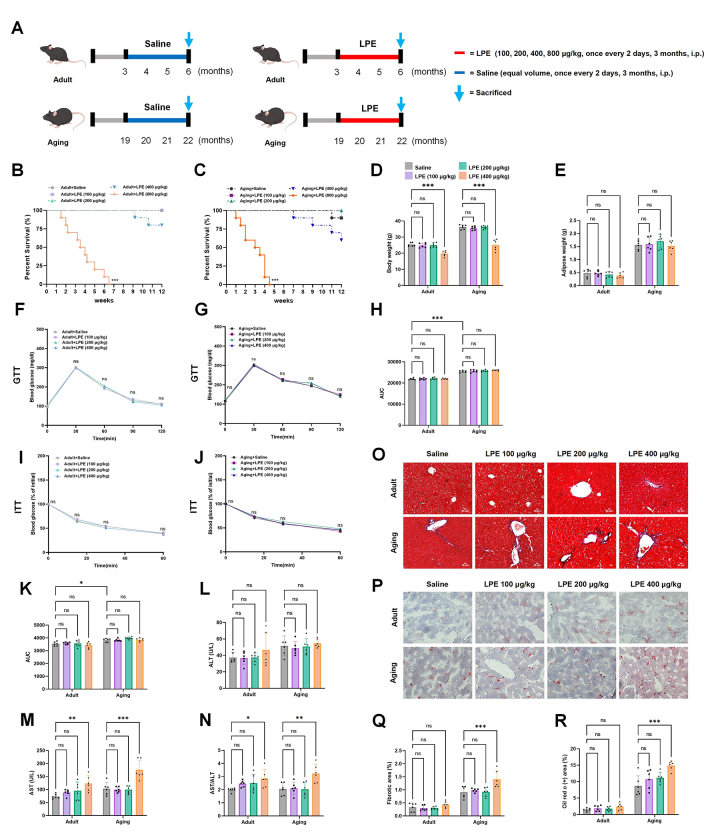


We tested four LPE treatment regimes in mice, including 100, 200, 400, and 800 μg/kg, delivered by intraperitoneal injection, once every two days for 3 months (Supplemental Fig. S1A). This enabled us to produce data for 3-months survival curves that showed that a dosage of 800 μg/kg was lethal to adult and aging mice, while exposure to 400 μg/kg led to a 20% and 40% mortality rate of adult and aging mice, respectively, within 3 months after LPE injection (Supplemental Fig. S1B, C). From this data, we wanted to explore the effects of LPE supplementation at 100, 200, and 400 μg/kg on various systems-level metabolic parameters, including body weight, adipose weight, GTT, and ITT in adult and aging mice. Body weight was comparable among the saline group, LPE_100 μg/kg_ group, and LPE_200 μg/kg_ group in adult mice and aging mice at 3 months after LPE injection, while LPE supplementation at 400 μg/kg led to significant weight loss in adult and aging mice, relative to saline group (without LPE treatment) (Supplemental Fig. S1D). Meanwhile, adipose weight, glucose tolerance and insulin tolerance were comparable across saline group, LPE_100 μg/kg_ group, LPE_200 μg/kg_ group and LPE_400 μg/kg_ group in adult and aging mice (Supplemental Fig. 1E–K).

Next, the effects of LPE treatment at 100, 200, and 400 μg/kg on liver damage were assessed by measures of serum ALT, AST, and AST/ALT levels, as well as hepatic Masson staining and Oil Red O staining. As shown, no significant differences were observed in ALT levels among the saline group, LPE_100 μg/kg_ group, LPE_200 μg/kg_ group, and LPE_400 μg/kg_ group in both adult and aging mice (Supplemental Fig. S1L). However, levels of AST and AST/ALT were significantly enhanced in adult and aging mice in the LPE_400 μg/kg_ group, compared with the saline treatment group (Supplemental Fig. S1M, N). Moreover, compared to the saline treatment group, a dosage of 400 μg/kg significantly increased fibrotic area and the number of lipid droplets in the liver tissue of aging mice, without any effects on the liver tissue of adult mice (Supplemental Fig. S1O–R). Thus, we surmised from our studies that the dosage of 200 μg/kg was safe for mice, without evidence of abnormal systematic metabolic characteristics and liver damage as measured through our assays.

### LPE treatment at 200 μg/kg protects against diastolic dysfunction and structural remodeling in aging hearts

**Fig. 6:**
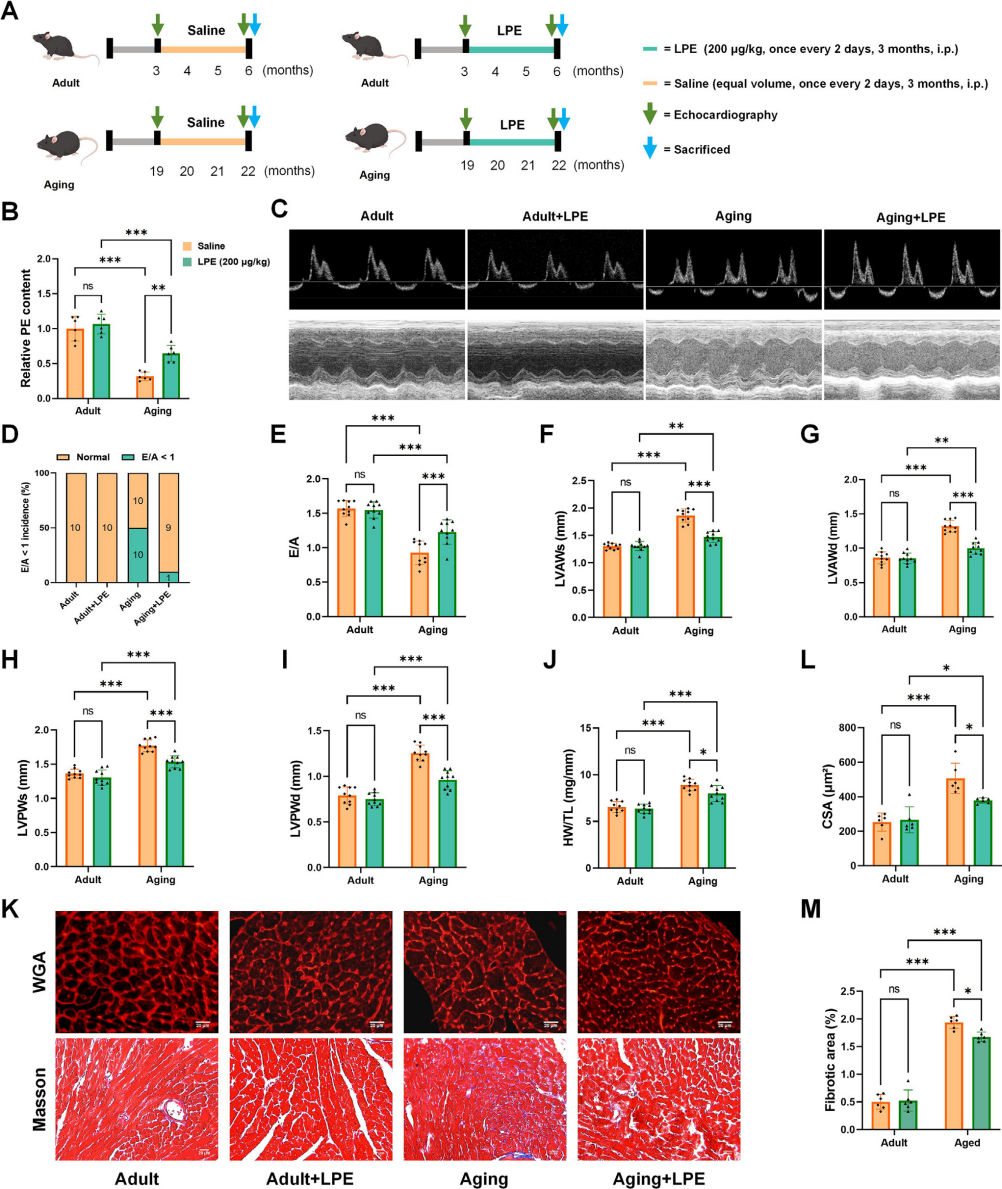
LPE treatment at 200 μg/kg protects against diastolic dysfunction and structural remodeling in aging hearts. Schematic diagram of the animal experiment design with supplementation of LPE at 200 μg/kg (A). Quantification of total mitochondrial PE in adult and aging mouse hearts with saline or LPE treatment (B). Representative left ventricular M-mode images and pulse-wave Doppler images (C). The proportion of mice with E/A < 1 (D). Assessment of cardiac function by echocardiography: E/A (E), LVAWs (F), LVAWd (G), LVPWs (H) and LVPWd (I). Quantification of HW/Tl (J). Representative images of WGA and Masson staining of ventricular tissues from adult and aging mice treated with saline or LPE (K). Quantification of cardiomyocytes CSA (L) and fibrotic area (M). LPE: lysophosphatidylethanolamine. All data were analyzed using two-way ANOVA. ∗*P* < 0.033, ∗∗*P* < 0.002, ∗∗∗*P* < 0.001. Sample sizes were n = 10 per group for echocardiography and HW/Tl evaluation, while sample sizes were n = 6 per group for analysis of PE content and pathological changes.

**Supplemental Figure S2:**
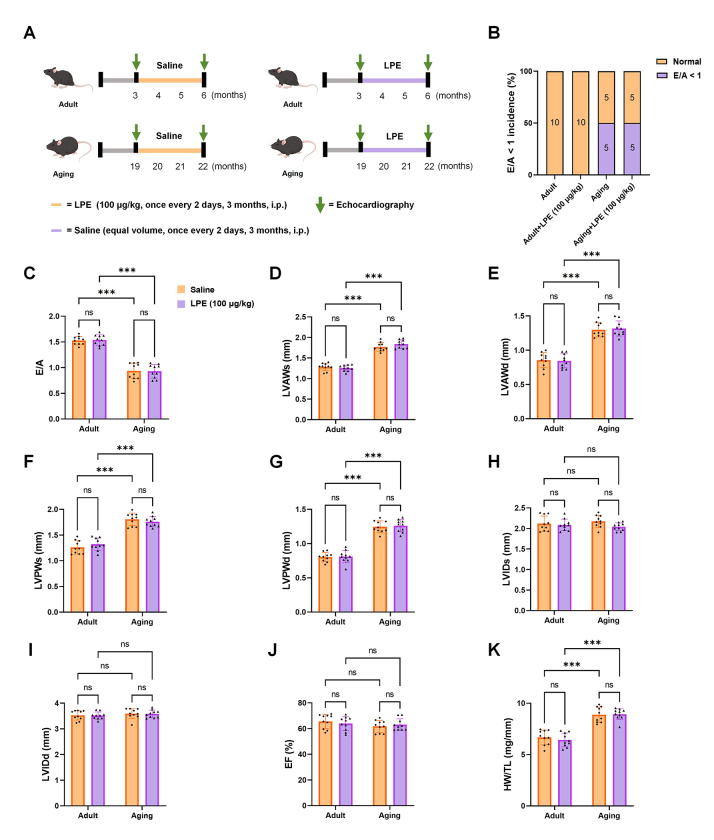


LPE supplementation at levels in mice that did not induce abnormal systemic metabolic characteristics, namely, at 100 μg/kg and 200 μg/kg, was tested to evaluate its potential for improving aging hearts. When aging mice were supplemented with LPE at 100 μg/kg, all echocardiographic parameters and cardiac function were comparable between aging mice without and with LPE treatment (Supplemental Fig. 2A–K). Next, we supplemented aging mice with 200 μg/kg LPE (Fig. 6A), we found that the content of PE significantly increased in the aging group with LPE supplementation, compared with the saline treatment in 22-month-old mice (Fig. 6B). The proportion of E/A < 1 was 10% in aging mice with LPE supplementation, which was notably lower than the incidence of E/A < 1 (50%) in the aging mice (Fig. 6C, D). The E/A ratio was significantly increased in aging mice with LPE treatment, compared with the aging mice receiving saline treatment (Fig. 6C, E). Furthermore, LPE treatment markedly reversed aging-induced the increase in LV wall thickness, measured by measuring LVAWs/d and LVPWs/d (Fig. 6C, F–I). The results indicate that aging-induced cardiac dysfunction in these mice could be alleviated by LPE treatment using our protocol. Also, compared with saline-treated aging mice, the ratio of HW to TL was decreased following LPE treatment in aging mice (Fig. 6J). Furthermore, WGA and Masson staining showed that aging-induced cardiac hypertrophy and enhanced fibrosis were both markedly ameliorated with LPE supplementation (Fig. 6K–M).

### LPE treatment rescued aging-induced mitochondrial respiration damage, morphological changes, and functional impairment of mitochondria

**Fig. 7:**
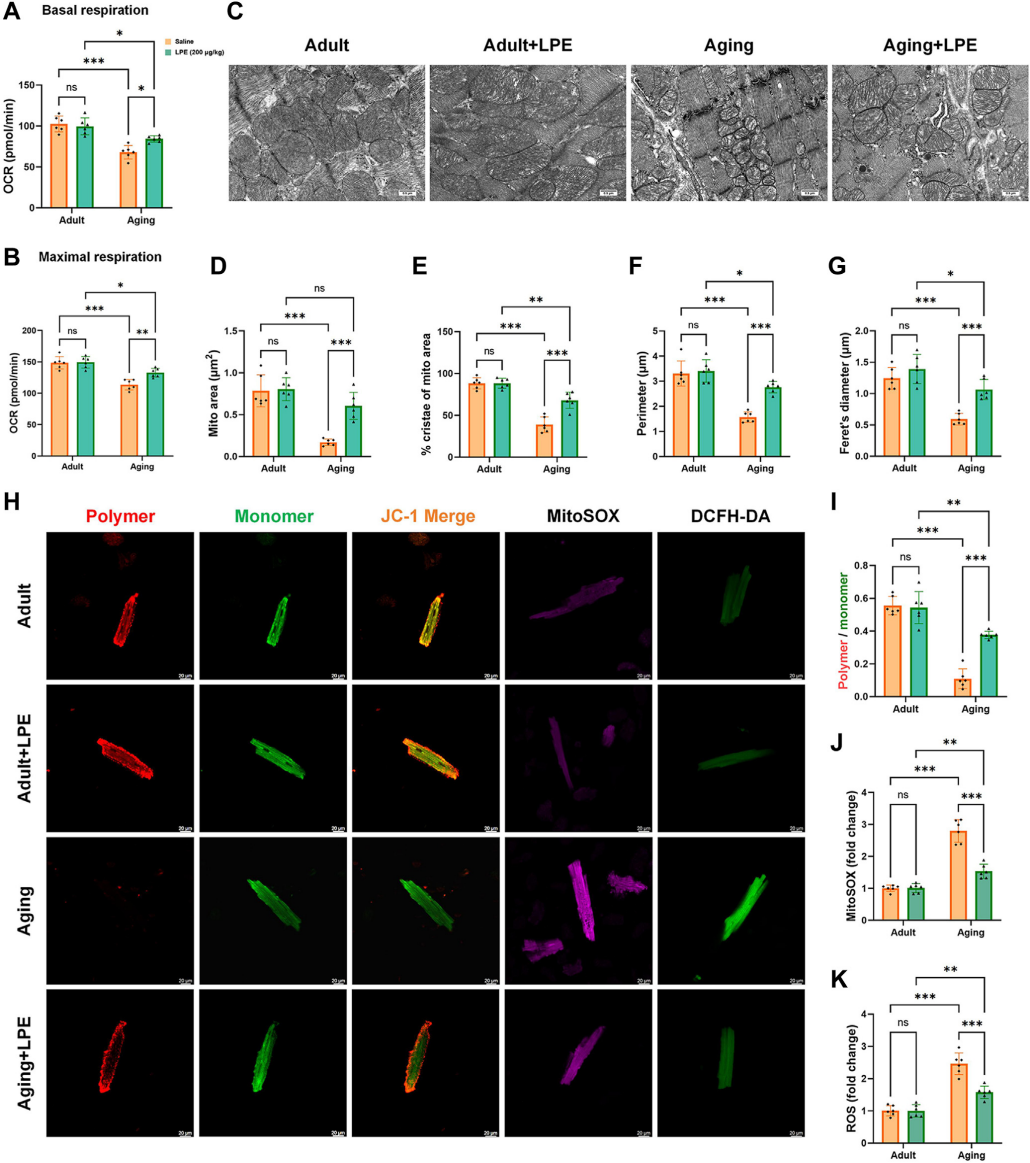
LPE treatment rescued aging-induced mitochondrial respiration damage, morphological changes and functional impairment of mitochondria. Quantitative analysis of basal respiration (A) and maximal respiration (B) of cardiomyocytes isolated from adult and aging mice with saline or LPE supplementation, measured using a Mito Stress Test kit. Representative TEM images of cardiac mitochondria in heart tissue sections from adult and aging mice following saline or LPE treatment (C). Quantification of the mitochondrial morphology: mitochondria area (μm^2^) (D), ratio of cristae-covered area to the whole mitochondria area (E), perimeter (μm) (F), and Feret's diameter (μm) (G). Representative images of mitochondrial membrane potential stained by JC-1 (*red* fluorescence for JC-1 polymer, green fluorescence for JC-1 monomers), MitoSOX-stained mitochondrial ROS (*purple* fluorescence) and DCFH-DA-stained cellular ROS production (*green* fluorescence) (H). Quantitative analysis of the ratio of JC-1 polymer to JC-1 monomer fluorescence intensity (I), MitoSox (J) and DCFH-DA (K). LPE: lysophosphatidylethanolamine. All data were analyzed using two-way ANOVA. ∗*P* < 0.033, ∗∗*P* < 0.002, ∗∗∗*P* < 0.001. Sample sizes were n = 6 per group.

As evidenced in the results from the Seahorse XFe96 Cell Mito Stress Tests, we found that LPE supplementation could ameliorate the reductions in basal respiration and maximal respiration in aging cardiomyocytes (Fig. 7A, B). Following this experiment, we next performed TEM to determine whether ultrastructural changes in the mitochondria of cells within the heart in mice are influenced by LPE treatment (Fig. 7C). The results showed that, in cells of the hearts of aging mice treated with LPE, there was evidence of improved mitochondrial surface area, cristae density, perimeter and Feret's diameter (Fig. 7D–G). Furthermore, compared with samples from aging mice treated with saline, the JC-1 polymers to monomers fluorescence ratios significantly increased in the cardiomyocytes of aging mice with LPE treatment (Fig. 7H, I). In aging mice with LPE supplementation, MitoSOX staining, and DCFH-DA assays revealed significant decreases in mitochondrial-specific ROS and intracellular total ROS production (Fig. 7H, J, K). Thus, these results indicate that LPE treatment could rescue the abnormal mitochondria features in the heart cells of aging mice.

### LPE treatment ameliorated the expression levels of key genes associated with mitochondrial homeostasis and metabolism in aging hearts

**Fig. 8:**
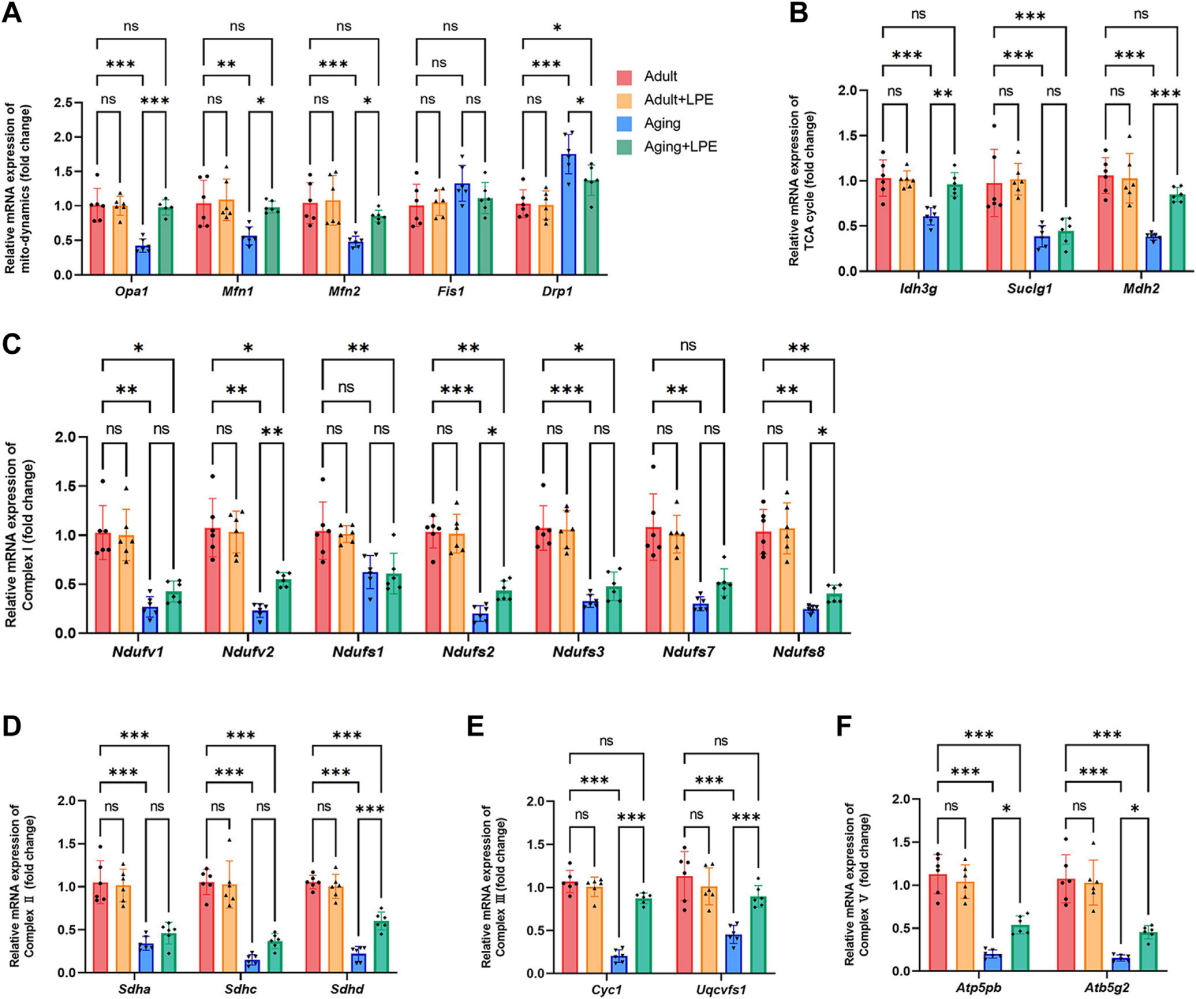
LPE treatment ameliorated the expression levels of key genes associated with mitochondrial homeostasis and metabolism in aging hearts. Steady-state mRNA levels of mitochondrial fusion-related genes (*Opa1*, *Mfn1* and *Mfn2*) and fission-related genes (*Fis1* and *Drp1*) are shown for samples (A). The mRNA levels of tricarboxylic acid (TCA) cycle-related genes (*Idh3g*, *Suclg1* and *Mdh2*) are shown (B). Quantitative analysis of the essential components of oxidative phosphorylation (OXPHOS): complex I (*Ndufv1*, *Ndufv2*, *Ndufs1*, *Ndufs2*, *Ndufs3*, *Ndufs7* and *Ndufs8*) (C), complex II (*Sdha*, *Sdhc* and *Sdhd*) (D), complex III (*Cyc1* and *Uqcrfs1*) (E) and complex V (*Atp5pb* and *Atp5g2*) are shown (F). LPE: lysophosphatidylethanolamine, All data were analyzed by two-way ANOVA. ∗*P* < 0.033, ∗∗*P* < 0.002, ∗∗∗*P* < 0.001. Sample sizes were n = 6 per group.

We analyzed mRNA levels of mitochondrial fusion genes *Opa1*, *Mfn1*, and *Mfn2* and found that these were significantly reduced in the aging mice, while levels of the mitochondrial fission gene *Drp1* were significantly increased, compared with the adult group (Fig. 8A). Nevertheless, we found no significant differences in *Fis1* expression between adult and aging mice (Fig. 8A). Additionally, LPE treatment resulted in a significant enhancement in *Opa1*, *Mfn1*, and *Mfn2* expression, as well as reduced *Drp1* expression levels in the aging hearts, compared with the saline treatment in aging mice (Fig. 8A). Genes associated with the TCA cycle, including *Idh3g*, *Suclg1*, and *Mdh2* were markedly downregulated in the aging heart (Fig. 8B). Moreover, LPE treatment significantly upregulated the gene expression of *Idh3g* and *Mdh2* in the aging mice, compared with saline-treated aging mice (Fig. 8B). Furthermore, the gene expression levels for OXPHOS complexes were measured, and our results revealed that LPE supplementation significantly improved aging-induced decreases in the expression levels for genes related to complex I (*Ndufv2*, *Ndufs2* and *Ndufs8*), complex II (*Sdhd*), complex III (*Cyc1* and *Uqcrfs1*) and complex V (*Atp5pb* and *Atp5g2*) (Fig. 8C–F). Therefore, these results demonstrate that LPE treatment influences the gene expression levels of mitochondrial genes in aging mice hearts.

## Discussion

In our current study, we have analyzed aged mice to find cardiac diastolic dysfunction and structural remodeling are associated with mitochondrial cristae remodeling, abnormal mitochondrial respiration, loss of mitochondrial membrane potential, and increases in mitochondrial ROS production in cells of the heart. We performed lipidomic analysis of cardiomyocyte mitochondria and discovered significant lipid composition changes in aging mice, most notably, a marked decrease in PE, compared to adult mice. The abnormal respiration and membrane potential features in mitochondria in cardiomyocyte cultures that were associated with PE deficiency were restored by LPE supplementation. Furthermore, we have found evidence that LPE supplementation in aging mice improved aging-induced cardiac diastolic dysfunction and structural remodeling, as well as ameliorating the impaired mitochondrial structure and function seen in aging mice, such as by increasing the cristae area in mitochondria, attenuating the loss of mitochondrial membrane potential, and reducing mitochondrial and intracellular ROS.

Myocardial mitochondrial dysfunction is reported as a molecular mechanism that underlies HFpEF (21, 22). Chaanine *et al.* examined the mitochondrial ultrastructure of cardiomyocytes from LV subepicardial tissues of elderly HFpEF patients and reported mitochondrial fragmentation, cristae destruction, vacuolar degeneration, and reduced mitochondrial area in those specimens (22). Additionally, Zhang *et al.* investigated mitochondrial morphology, dynamics, and function in a Dahl salt-induced rat HFpEF model and employed multi-omics and GO analyses to report that mitochondrial fission and the inflammatory response were top biological processes contributing to cardiomyocyte stiffening in HFpEF (23). Hypertrophy and fibrosis are key pathological features associated with cardiac diastolic dysfunction (24, 25, 26). Moreover, several studies have provided strong evidence that mitochondrial dysfunction is the primary mechanism for myocardial hypertrophy and fibrosis (26, 27, 28). Consistent with these previous reports, our current findings show that aging mice with reduced diastolic function and structural remodeling exhibit myocardial mitochondrial dysfunction, as measured by abnormal mitochondrial respiration and morphology, reduced membrane potential, and alterations to gene expression profiles that underlie mitochondrial functions. Furthermore, we found that such changes were associated with alterations in mitochondrial lipid composition and metabolites. However, whether alterations to lipid composition and metabolites in mitochondria of aging hearts are causative for mitochondrial injury and whether restoration of lipid metabolism in cardiomyocytes could influence cardiac aging and disease all remain better characterized.

Mitochondrial function and membrane integrity are both critical for the maintenance of the high-energy requirements of cardiomyocytes (29, 30). The protein complexes of the respiratory chain are predominantly localized in the cristae membranes, and alterations in these membranes affect respiratory efficiency (31). It is recognized that the predominant phospholipid species of mitochondrial membranes are PE and PC (32), and these are vital for maintaining membrane integrity as well as mitochondrial function (18). Indeed, deletion of PE production pathways in mice causes embryonic lethality and mitochondrial defects (33, 34). Burstein *et al.* investigated that specific remodeling of mitochondrial phospholipid dynamics in senescence yeast cells led to a significant decrease in the relative levels of PE in the mitochondrial membrane lipidome (35). Current studies have shown that the content of PE in mitochondria is markedly reduced in senescent cells of the heart, muscles, and brain (36, 37, 38), while fragmented mitochondria were found to be increased, concomitant with inhibition in mitochondrial fusion with age (39, 40). In addition to its vital inclusion as a mitochondrial membrane component, PE is also required for the biogenesis of mitochondrial proteins Opa1/Mgm1, which is directly related to mitochondrial fusion (20). Chan and Joshi *et al.* reported that deficiencies in mitochondrial PE led to decreased levels of S-Mgm1 protein and mitochondrial fusion (41, 42). Based on these observations, the depletion of PE is essential to a molecular mechanism for the inhibition of mitochondrial fusion in homeostasis, aging, and disease.

In the current study, we performed lipidomic analysis on the mitochondria of the hearts and discovered that PE content was significantly decreased in aging (22-month-old) mice, compared with adult (3-month-old) mice. PE is synthesized through two pathways: the Kennedy pathway and a pathway involving decarboxylation of PS via the mitochondrial PSD (encoded by the *PISD* gene) (43, 44). The CDP-ethanolamine pathway involves three enzymatic steps which take place within the endoplasmic reticulum (43, 45), catalyzed by ETNK1, PCYT2, and SELENOI. Additionally, the mitochondrial intermembrane protein PRELID3/UPS2 is involved in the transport of PS from the outer mitochondrial membrane to the inner mitochondrial membrane. Ostensibly, deletion of UPS2 leads to reduced mitochondrial PE levels (46, 47). In the present study, we found the mRNA level of *Pisd* was significantly decreased in aged mice. We further confirmed that cardiac *Pisd* was reduced in 19-month-old mice. Moreover, *Pisd* knockdown in cardiomyocytes led to mitochondrial dysfunction. Collectively, we speculated that the time point at which cardiac PE level decline is likely at 19 months of age, and mitochondrial dysfunction in aged mice was attributable to PE deficiency.

PE can also be produced via the acylation of LPE by acyl-CoA transferase enzymes MBOAT1, LPCAT3, and LPCAT4 (48, 49). Although the LPE acylation pathway is known to contribute only in a minor capacity to cellular PE compared to synthesis via the Kennedy and PSD pathways, LPE supplementation could enhance the levels of PE in mitochondria and improve the morphology and biological function of resident mitochondria (50, 51), both in the in vivo and in vitro contexts.

Thus, we examined the effects of supplementation with LPE for 3 months in both adult and aging mice on mitochondrial morphology and function. We further confirmed that LPE supplementation could partially restore the mRNA expression levels of genes associated with mitochondrial dynamic functions in aging mice through enhancing PE content, such as the fusion gene *Opa1*. In addition, our TEM studies showed that the decreases in mitochondria size and deterioration of mitochondrial cristae in aging mice were ameliorated by LPE treatment. Furthermore, LPE treatment partially restored the expression of genes related to OXPHOS (respiration chain subunits) and the TCA cycle in aging mice, indicating that maintaining the integrity of mitochondrial membrane structure can, at least in part, restore such functions.

There are active efforts in the world to develop clinical pharmacological therapies for heart failure that aim to restore mitochondrial function (52). Our findings in this study are consistent with the notion that LPE supplementation promotes mitochondrial fusion, as well as improve mitochondrial function by enhancing PE content in mitochondria which, in turn, reduces apoptosis and possibly increases cell proliferation (51, 53, 54). Yamamoto *et al.* reported that increased LPE may play a pathological role in the development of nonalcoholic fatty liver disease by inhibiting lipolysis and fatty acid biosynthesis (55). Furthermore, a previous study found that elevated PE was associated with occlusive arterial disease in humans (56). On the basis of these observations, we conclude that the timing, dosage, safety, and efficacy of LPE must be carefully regulated to achieve beneficial effects in aging mice and to avoid unwanted physiological consequences.

## Conclusion

Aging mice with cardiac diastolic dysfunction showed reductions in mitochondrial PE, abnormal mitochondrial respiration, disruption of mitochondrial inner structures, and decreased mitochondrial membrane potential. In the future, an optimal LPE treatment could be developed to increase the content of PE in the mitochondria of aging mice, restore mitochondrial structure, maintain the dynamic balance between mitochondrial fusion and fission, as well as decrease mitochondrial and intracellular ROS, so as to ameliorate aging-induced cardiac diastolic dysfunction.

## Data availability

All data described are included in the manuscript and the supplementary material. The data presented in this study are available on request from the corresponding authors.

## Supplemental data

This article contains supplemental data.

## Conflict of interest

The authors declare that they have no conflicts of interest with the contents of this article.

## References

[1] Campbell P., Rutten F.H., Lee M.M., Hawkins N.M., Petrie M.C. (2024). Heart failure with preserved ejection fraction: everything the clinician needs to know. Lancet (London, England).

[2] Pfeffer M.A., Shah A.M., Borlaug B.A. (2019). Heart failure with preserved ejection fraction in perspective. Circ. Res..

[3] Dunlay S.M., Roger V.L., Redfield M.M. (2017). Epidemiology of heart failure with preserved ejection fraction. Nat. Rev. Cardiol..

[4] Guo J., Huang X., Dou L., Yan M., Shen T., Tang W. (2022). Aging and aging-related diseases: from molecular mechanisms to interventions and treatments. Signal Transduct. Target Ther..

[5] Dai D.F., Rabinovitch P.S., Ungvari Z. (2012). Mitochondria and cardiovascular aging. Circ. Res..

[6] Dai D.F., Santana L.F., Vermulst M., Tomazela D.M., Emond M.J., MacCoss M.J. (2009). Overexpression of catalase targeted to mitochondria attenuates murine cardiac aging. Circulation.

[7] Poznyak A.V., Kirichenko T.V., Borisov E.E., Shakhpazyan N.K., Kartuesov A.G., Orekhov A.N. (2022). Mitochondrial implications in cardiovascular aging and diseases: the specific role of mitochondrial dynamics and shifts. Int. J. Mol. Sci..

[8] Ngo J., Choi D.W., Stanley I.A., Stiles L., Molina A.J.A., Chen P.H. (2023). Mitochondrial morphology controls fatty acid utilization by changing CPT1 sensitivity to malonyl-CoA. EMBO J..

[9] Lesnefsky E.J., Chen Q., Hoppel C.L. (2016). Mitochondrial metabolism in aging heart. Circ. Res..

[10] Li X., Wang J., Wang L., Gao Y., Feng G., Li G. (2022). Lipid metabolism dysfunction induced by age-dependent DNA methylation accelerates aging. Signal Transduct. Target Ther..

[11] Liu X., Hartman C.L., Li L., Albert C.J., Si F., Gao A. (2021). Reprogramming lipid metabolism prevents effector T cell senescence and enhances tumor immunotherapy. Sci. Transl Med..

[12] Wu J., Bu D., Wang H., Shen D., Chong D., Zhang T. (2023). The rhythmic coupling of Egr-1 and Cidea regulates age-related metabolic dysfunction in the liver of male mice. Nat. Commun..

[13] Cho Y.K., Yoon Y.C., Im H., Son Y., Kim M., Saha A. (2022). Adipocyte lysoplasmalogenase TMEM86A regulates plasmalogen homeostasis and protein kinase A-dependent energy metabolism. Nat. Commun..

[14] Flameng W., Borgers M., Daenen W., Stalpaert G. (1980). Ultrastructural and cytochemical correlates of myocardial protection by cardiac hypothermia in man. J. Thorac. Cardiovasc. Surg..

[15] Ackers-Johnson M., Li P.Y., Holmes A.P., O'Brien S.M., Pavlovic D., Foo R.S. (2016). A simplified, langendorff-free method for concomitant isolation of viable cardiac myocytes and nonmyocytes from the adult mouse heart. Circ. Res..

[16] Matyash V., Liebisch G., Kurzchalia T.V., Shevchenko A., Schwudke D. (2008). Lipid extraction by methyl-tert-butyl ether for high-throughput lipidomics. J. Lipid Res..

[17] Tasseva G., Bai H.D., Davidescu M., Haromy A., Michelakis E., Vance J.E. (2013). Phosphatidylethanolamine deficiency in Mammalian mitochondria impairs oxidative phosphorylation and alters mitochondrial morphology. J. Biol. Chem..

[18] Horvath S.E., Daum G. (2013). Lipids of mitochondria. Prog. Lipid Res..

[19] Martensson C.U., Doan K.N., Becker T. (2017). Effects of lipids on mitochondrial functions. Biochim. Biophys. Acta Mol. Cell Biol. Lipids.

[20] Zhang Q., Tamura Y., Roy M., Adachi Y., Iijima M., Sesaki H. (2014). Biosynthesis and roles of phospholipids in mitochondrial fusion, division and mitophagy. Cell Mol. Life Sci..

[21] Lozhkin A., Vendrov A.E., Ramos-Mondragon R., Canugovi C., Stevenson M.D., Herron T.J. (2022). Mitochondrial oxidative stress contributes to diastolic dysfunction through impaired mitochondrial dynamics. Redox Biol..

[22] Deng Y., Xie M., Li Q., Xu X., Ou W., Zhang Y. (2021). Targeting mitochondria-inflammation circuit by beta-hydroxybutyrate mitigates HFpEF. Circ. Res..

[23] Zhang W., Zhang H., Yao W., Li L., Niu P., Huo Y. (2020). Morphometric, hemodynamic, and multi-omics analyses in heart failure rats with preserved ejection fraction. Int. J. Mol. Sci..

[24] Sun S.N., Ni S.H., Li Y., Liu X., Deng J.P., Chen Z.X. (2021). G-MDSCs promote aging-related cardiac fibrosis by activating myofibroblasts and preventing senescence. Cell Death Dis.

[25] Biernacka A., Frangogiannis N.G. (2011). Aging and cardiac fibrosis. Aging Dis..

[26] Nakamura M., Sadoshima J. (2018). Mechanisms of physiological and pathological cardiac hypertrophy. Nat. Rev. Cardiol..

[27] Dai D.F., Rabinovitch P.S. (2009). Cardiac aging in mice and humans: the role of mitochondrial oxidative stress. Trends Cardiovasc. Med..

[28] Sawyer D.B., Siwik D.A., Xiao L., Pimentel D.R., Singh K., Colucci W.S. (2002). Role of oxidative stress in myocardial hypertrophy and failure. J. Mol. Cell Cardiol..

[29] Kuzmicic J., Del Campo A., Lopez-Crisosto C., Morales P.E., Pennanen C., Bravo-Sagua R. (2011). [Mitochondrial dynamics: a potential new therapeutic target for heart failure]. Rev. Esp Cardiol..

[30] Vasquez-Trincado C., Garcia-Carvajal I., Pennanen C., Parra V., Hill J.A., Rothermel B.A. (2016). Mitochondrial dynamics, mitophagy and cardiovascular disease. J. Physiol..

[31] Colina-Tenorio L., Horten P., Pfanner N., Rampelt H. (2020). Shaping the mitochondrial inner membrane in health and disease. J. Intern. Med..

[32] van Meer G., Voelker D.R., Feigenson G.W. (2008). Membrane lipids: where they are and how they behave. Nat. Rev. Mol. Cell Biol..

[33] Fullerton M.D., Hakimuddin F., Bakovic M. (2007). Developmental and metabolic effects of disruption of the mouse CTP:phosphoethanolamine cytidylyltransferase gene (Pcyt2). Mol. Cell Biol..

[34] Steenbergen R., Nanowski T.S., Beigneux A., Kulinski A., Young S.G., Vance J.E. (2005). Disruption of the phosphatidylserine decarboxylase gene in mice causes embryonic lethality and mitochondrial defects. J. Biol. Chem..

[35] Burstein M.T., Titorenko V.I. (2014). A mitochondrially targeted compound delays aging in yeast through a mechanism linking mitochondrial membrane lipid metabolism to mitochondrial redox biology. Redox Biol..

[36] Papsdorf K., Brunet A. (2019). Linking lipid metabolism to chromatin regulation in aging. Trends Cell Biol..

[37] Paradies G., Ruggiero F.M., Dinoi P. (1992). Decreased activity of the phosphate carrier and modification of lipids in cardiac mitochondria from senescent rats. Int. J. Biochem..

[38] Zappelli E., Daniele S., Ceccarelli L., Vergassola M., Ragni L., Mangano G. (2022). alpha-Glyceryl-phosphoryl-ethanolamine protects human hippocampal neurons from ageing-induced cellular alterations. Eur. J. Neurosci..

[39] Erchova I., Sun S., Votruba M. (2021). A perspective on accelerated aging caused by the genetic deficiency of the metabolic protein, OPA1. Front. Neurol..

[40] Wang C., Yang K., Liu X., Wang S., Song M., Belmonte J.C.I. (2023). MAVS antagonizes human stem cell senescence as a mitochondrial stabilizer. Research (Wash D C).

[41] Chan E.Y., McQuibban G.A. (2012). Phosphatidylserine decarboxylase 1 (Psd1) promotes mitochondrial fusion by regulating the biophysical properties of the mitochondrial membrane and alternative topogenesis of mitochondrial genome maintenance protein 1 (Mgm1). J. Biol. Chem..

[42] Joshi A.S., Thompson M.N., Fei N., Huttemann M., Greenberg M.L. (2012). Cardiolipin and mitochondrial phosphatidylethanolamine have overlapping functions in mitochondrial fusion in Saccharomyces cerevisiae. J. Biol. Chem..

[43] Kennedy E.P., Weiss S.B. (1956). The function of cytidine coenzymes in the biosynthesis of phospholipides. J. Biol. Chem..

[44] Schuiki I., Daum G. (2009). Phosphatidylserine decarboxylases, key enzymes of lipid metabolism. IUBMB Life.

[45] Fu G., Guy C.S., Chapman N.M., Palacios G., Wei J., Zhou P. (2021). Metabolic control of T(FH) cells and humoral immunity by phosphatidylethanolamine. Nature.

[46] Tamura Y., Sesaki H., Endo T. (2014). Phospholipid transport via mitochondria. Traffic.

[47] Kojima R., Kakimoto Y., Furuta S., Itoh K., Sesaki H., Endo T. (2019). Maintenance of cardiolipin and crista structure requires cooperative functions of mitochondrial dynamics and phospholipid transport. Cell Rep..

[48] Hishikawa D., Shindou H., Kobayashi S., Nakanishi H., Taguchi R., Shimizu T. (2008). Discovery of a lysophospholipid acyltransferase family essential for membrane asymmetry and diversity. Proc. Natl. Acad. Sci. U. S. A..

[49] St Germain M., Iraji R., Bakovic M. (2022). Phosphatidylethanolamine homeostasis under conditions of impaired CDP-ethanolamine pathway or phosphatidylserine decarboxylation. Front. Nutr..

[50] Montero-Bullon J.F., Melo T., Ferreira R., Padrao A.I., Oliveira P.A., Domingues M.R.M. (2019). Exercise training counteracts urothelial carcinoma-induced alterations in skeletal muscle mitochondria phospholipidome in an animal model. Sci. Rep..

[51] Liu N., Huang L., Xu H., He X., He X., Cao J. (2023). Phosphatidylserine decarboxylase downregulation in uric acid-induced hepatic mitochondrial dysfunction and apoptosis. MedComm..

[52] Brown D.A., Perry J.B., Allen M.E., Sabbah H.N., Stauffer B.L., Shaikh S.R. (2017). Expert consensus document: mitochondrial function as a therapeutic target in heart failure. Nat. Rev. Cardiol..

[53] Keckesova Z., Donaher J.L., De Cock J., Freinkman E., Lingrell S., Bachovchin D.A. (2017). LACTB is a tumour suppressor that modulates lipid metabolism and cell state. Nature.

[54] Zhao T., Goedhart C.M., Sam P.N., Sabouny R., Lingrell S., Cornish A.J. (2019). PISD is a mitochondrial disease gene causing skeletal dysplasia, cataracts, and white matter changes. Life Sci. Alliance.

[55] Yamamoto Y., Sakurai T., Chen Z., Inoue N., Chiba H., Hui S.P. (2022). Lysophosphatidylethanolamine affects lipid accumulation and metabolism in a human liver-derived cell line. Nutrients.

[56] Kunz F., Stummvoll W. (1971). Plasma phosphatidylethanolamine--a better indicator in the predictability of atherosclerotic complications?. Atherosclerosis.

