# Supplementary material for: Lysophosphatidylethanolamine improves diastolic dysfunction by alleviating mitochondrial injury in the aging heart
Source: J Lipid Res. 2024 Nov 22;66(1):100713. doi: 10.1016/j.jlr.2024.100713 (PMC11719853; doi:10.1016/j.jlr.2024.100713)
Supplement: Supplemental Material [file mmc1.docx]

**Supplemental Material**

**Lysophosphatidylethanolamine improves diastolic dysfunction by alleviating mitochondrial injury in aging heart**

Guiwen Xu, MBBS ^1, #^, Wei Xiao, MBBS ^1, #^, Pengqi Sun, MBSS ^1^, Yuanjun Sun, MBBS ^2^, Xinyu Yang, MBBS ^1^, Xiaomeng Yin, PhD ^2, *^, Yang Liu, PhD ^1, *^

^#^These authors contributed equally

^1^Institute of Cardiovascular Diseases, the First Affiliated Hospital of Dalian Medical University, Dalian, China; ^2^Department of Cardiology, the First Affiliated Hospital of Dalian Medical University, Dalian, 116011, China.

**Running title:** Mitochondrial lipid metabolism in aging heart

* Corresponding Authors:

Yang Liu, Institute of Cardiovascular Diseases, the First Affiliated Hospital of Dalian Medical University, No 222 Zhongshan Rd, Dalian, China 0086; Email: liuyang19831119@163.com.

Xiaomeng Yin, Department of Cardiology, First Affiliated Hospital of Dalian Medical University, No 222 Zhongshan Rd, Dalian, China 0086; E-mail: dr.yinxm@126.com;

**FIGURE LEGEND**

**Supplemental Figure 1: Metabolic phenotypes and safety characteristics of LPE exposure in treated mice**

Diagrammatic representation of the animal experimental design with different doses (100, 200, 400 and 800 µg/kg) of LPE supplementation (**A**). Survival curve of adult mice (**B**) and aging mice (**C**) treated with LPE at 100, 200, 400 and 800 µg/kg. Body weight (**D**) and adipose weight (**E**) of adult or aging mice with saline or LPE (100, 200 and 400 µg/kg) treatment. Glucose tolerance test (GTT) and insulin tolerance test (ITT) for adult mice (**F** and **I**) and aging mice (**G** and **J**) with saline or LPE (100, 200 and 400 µg/kg) supplementation. Area under the curve (AUC) derived from blood glucose levels during GTT (**H**) and ITT (**K**). Quantification of alanine transaminase (ALT), aspartate transaminase (AST) and AST/ALT levels in serum, as markers for hepatocellular injury (**L-N**). Representative images of Masson and oil red o staining of liver slides (**O** and **P**). Quantification of the fibrotic area (**Q**) and lipid droplet area (**R**). Survival curves were analyzed using Mantel-Cox log-rank tests, n=10 per group. Statistical analyses for (D)-(N) and (Q)-(R) were performed by two-way ANOVA, n=6 per group. *P<0.033, **P<0.002, ***P<0.001.

**Supplemental Figure 2: Effects of LPE at 100μg/kg on cardiac function in adult and aging mice**

The animal experimental design with LPE supplementation at 100μg/kg of study is presented in (**A**). Fraction of mice with E/A < 1 (**B**). Assessment of cardiac function by echocardiography: E/A (**C**), LVAWs (**D**), LVAWd (**E**), LVPWs (**F**), LVPWd (**G**), LVIDs (**H**), LVIDd (**I**) and EF% (**J**). Quantification of HW/TL (**K**). All data were analyzed using two-way ANOVA. *P<0.033, **P<0.002, ***P<0.001. Sample sizes were n=10 per group.

**Supplemental Table 1: Sequences of mouse primes for Real-time PCR**

| **Gene** |  | **Primer** |
| --- | --- | --- |
| *Actb* | Forward | 5’ - CATTGCTGACAGGATGCAGAAGG - 3’ |
|  | Reverse | 5’ - TGCTGGAAGGTGGACAGTGAGG - 3’ |
| *Atp5g2* | Forward | 5’ - TGCAGTGGAGTTGAAGCGACCA - 3’ |
|  | Reverse | 5’ - GCTGGTTTGGAAACTGCGGCTA - 3’ |
| *Atp5pb* | Forward | 5’ - GGTTCAGAAGCGCCATTACCTC - 3’ |
|  | Reverse | 5’ - GTTCTTCCTCCTTGCGACGCAT - 3’ |
| *Cyc1* | Forward | 5’ - CCATCTACACAGAAGTCTTGGAG - 3’ |
|  | Reverse | 5’ - GCGTTTTCGATGGTCATGCTCTG - 3’ |
| *Drp1* | Forward | 5’ - GCGAACCTTAGAATCTGTGGACC - 3’ |
|  | Reverse | 5’ - CAGGCACAAATAAAGCAGGACGG - 3’ |
| *Etnk1* | Forward | 5’ - CTGTTCACAGATGGGATCACAA - 3’ |
|  | Reverse | 5’ - CGCGGAAACTTTTCACTTCCTC - 3’ |
| *Fis1* | Forward | 5’ - GCTGGTTCTGTGTCCAAGAGCA - 3’ |
|  | Reverse | 5’ - GACATAGTCCCGCTGTTCCTCT - 3’ |
| *Idh3g* | Forward | 5’ - ACTCCTATGCCACTTCCATCCG - 3’ |
|  | Reverse | 5’ - CGAATGATGTCCTGGATGGCTTG - 3’ |
| *Lpcat3* | Forward | 5’ - CCATCTCTTCCACACCTTCACG - 3’ |
|  | Reverse | 5’ - GGATGAGGAACTGAAGCACGAC - 3’ |
| *Lpcat4* | Forward | 5’ - CGTGCATGAGTTACACCTCTCC - 3’ |
|  | Reverse | 5’ - CAGTGAGACCTGCTACTTGGAG - 3’ |
| *Mboat1* | Forward | 5’ - GCCTTTCGTCATGTTGGCAGTC - 3’ |
|  | Reverse | 5’ - TTGGACTCCGAGACTGCCTTTG - 3’ |
| *Mdh2* | Forward | 5’ - TCACTCCTGCTGAAGAACAGCC - 3’ |
|  | Reverse | 5’ - CCTTTGAGGCAATCTGGCAACTG - 3’ |
| *Mfn1* | Forward | 5’ - CCAGGTACAGATGTCACCACAG - 3’ |
|  | Reverse | 5’ - TTGGAGAGCCGCTCATTCACCT - 3’ |
| *Mfn2* | Forward | 5’ - GTGGAATACGCCAGTGAGAAGC - 3’ |
|  | Reverse | 5’ - CAACTTGCTGGCACAGATGAGC - 3’ |
| *Ndufs1* | Forward | 5’ - GTGGATGCTGAAGCCTTAGTAGC - 3’ |
|  | Reverse | 5’ - GGAACGTAAGTCTGTACCAGCTC - 3’ |
| *Ndufs2* | Forward | 5’ - CGTTTACGACCAGGTGGAGTTTG - 3’ |
|  | Reverse | 5’ - GAGGCATCTTGTTCAGACACTGC - 3’ |
| *Ndufs3* | Forward | 5’ - CTGACTTGACGGCAGTGGATGT - 3’ |
|  | Reverse | 5’ - GTGTCAGCTCATCTGCATAGGTC - 3’ |
| *Ndufs7* | Forward | 5’ - GGCTGAGTATGTGGTGACCAAG - 3’ |
|  | Reverse | 5’ - AGCCATGTGCATCATCTCCACG - 3’ |
| *Ndufs8* | Forward | 5’ - GGACAAAGCCTTCATAGCAGCG - 3’ |
|  | Reverse | 5’ - CTGTCCACATCAGAATCCGAGC - 3’ |
| *Ndufv1* | Forward | 5’ - GTGGAGGAAGAGATGTCTGTGC - 3’ |
|  | Reverse | 5’ - ATGAGCCACCAGGAATCACAGC - 3’ |
| *Opa1* | Forward | 5’ - TCTCAGCCTTGCTGTGTCAGAC - 3’ |
|  | Reverse | 5’ - TTCCGTCTCTAGGTTAAAGCGCG - 3’ |
| *Pcyt2* | Forward | 5’ - GGAAGACAGAGATTGTACCCGAC - 3’ |
|  | Reverse | 5’ - GCACAATCAGGTCTGTAGTGAGG - 3’ |
| *Pisd* | Forward | 5’ - TGACTGGACCATCTCACATCGG - 3’ |
|  | Reverse | 5’ - CTCCAGTTAGGACTACACGCTC - 3’ |
| *Prelid3b* | Forward | 5’ - CCTCTGGAAAGTTGCACAGCCA - 3’ |
|  | Reverse | 5’ - GGTCAACTACAGAATGCTCCTGC - 3’ |
| *Ptdss1* | Forward | 5’ - CAGCAAGTGGAGGACATCACCA - 3’ |
|  | Reverse | 5’ - CCTCTCCAGATGTTGTCTTCCG - 3’ |
| *Ptdss2* | Forward | 5’ - CTACGACGATGGCACTAACACC - 3’ |
|  | Reverse | 5’ - GTGTTGTAGGCTGTATCCTGAGG - 3’ |
| *Sdha* | Forward | 5’ - GAGATACGCACCTGTTGCCAAG - 3’ |
|  | Reverse | 5’ - GGTAGACGTGATCTTTCTCAGGG - 3’ |
| *Sdhc* | Forward | 5’ - TGCTCCTTTGGGAACCACAGCT - 3’ |
|  | Reverse | 5’ - GCAAACGGACAGTGCCATAGGA - 3’ |
| *Sdhd* | Forward | 5’ - GGTTGTCAGTGTTCTGCTCTTGG - 3’ |
|  | Reverse | 5’ - GTCGGTAACCACTTGTCCAAGG - 3’ |
| *Selenoi* | Forward | 5’ - GCTTTGGATACCAACCCACTCTC - 3’ |
|  | Reverse | 5’ - GGTCGAAGTATGTCAGGAGTAGG - 3’ |
| *Suclg1* | Forward | 5’ - GTCTTACACAGCCTCTCGGAAAC - 3’ |
|  | Reverse | 5’ - ACTCCAAAGCCTGCTGACTGTG - 3’ |
| *Uqcrfs1* | Forward | 5’ - CCTGTTCTGGATGTGAAGCGAC - 3’ |
|  | Reverse | 5’ - AGAGAAGTCGGGCACCTTGACA - 3’ |

**Supplemental Table 2: Sequences of human primes for Real-time PCR**

| **Gene** |  | **Primer** |
| --- | --- | --- |
| *ACTB* | Forward | 5’ - CACCATTGGCAATGAGCGGTTC - 3’ |
|  | Reverse | 5’ - AGGTCTTTGCGGATGTCCACGT - 3’ |
| *DRP1* | Forward | 5’ - GATGCCATAGTTGAAGTGGTGAC - 3’ |
|  | Reverse | 5’ - CCACAAGCATCAGCAAAGTCTGG - 3’ |
| *FIS1* | Forward | 5’ - CAAGGAACTGGAGCGGCTCATT - 3’ |
|  | Reverse | 5’ - GGACACAGCAAGTCCGATGAGT - 3’ |
| *MFN1* | Forward | 5’ - GGTGAATGAGCGGCTTTCCAAG - 3’ |
|  | Reverse | 5’ - TCCTCCACCAAGAAATGCAGGC - 3’ |
| *MFN2* | Forward | 5’ - ATTGCAGAGGCGGTTCGACTCA - 3’ |
|  | Reverse | 5’ - TTCAGTCGGTCTTGCCGCTCTT - 3’ |
| *OPA1* | Forward | 5’ - GTGGTTGGAGATCAGAGTGCTG - 3’ |
|  | Reverse | 5’ - GAGGACCTTCACTCAGAGTCAC - 3’ |
| *PISD* | Forward | 5’ - ATCACTACCGCAACCTCAGCGA - 3’ |
|  | Reverse | 5’ - TACCTGCTCCACCTCACAGTTC - 3’ |
